# A Unified Evaluation Protocol and Late-Fusion System for Monocular Per-Object Distance Estimation in Indoor Scenes

**DOI:** 10.3390/s26165013

**Published:** 2026-08-07

**Authors:** Adnan Ali, Yu Jun

**Affiliations:** Department of Automation, University of Science and Technology of China, Hefei 230026, China; aliadnan10@mail.ustc.edu.cn

**Keywords:** monocular depth estimation, per-object distance estimation, evaluation protocol, bounding box distance aggregation

## Abstract

Monocular per-object distance estimation aims to predict a metric distance for each detected object from a single RGB image. Although monocular dense depth estimation and monocular 3D object detection are well studied, indoor object-level distance estimation remains weakly standardized. In dense depth-based pipelines, object distance is commonly obtained by combining object detection with depth prediction and aggregating depth values within a region of interest. However, existing approaches differ in region selection, detection source, and aggregation strategy, limiting comparability across methods. This paper proposes a unified, deployment-aligned evaluation protocol for dense depth-based pipelines, where both predicted and reference distances are computed within the same predicted bounding box. This formulation standardizes region-of-interest selection, removes dependence on ground-truth boxes at inference time, and enables consistent evaluation across late-fusion methods. The framework integrates a curriculum-trained Depth Anything V2 ViT-S model for metric depth estimation with a YOLO11n detector for object localization. Under the same-box evaluation protocol on SUN RGB-D, the Depth Anything V2 ViT-S backbone achieves object-wise distance estimation accuracy of MAE = 0.1286 m, RMSE = 0.1817 m, AbsRel = 0.0662, and $\delta_{1}$ = 0.9785 using mean aggregation over valid box depths. Scaling the backbone from ViT-S to ViT-L further improves performance to MAE = 0.1059 m, RMSE = 0.1484 m, AbsRel = 0.0553, and $\delta_{1}$ = 0.9898, corresponding to approximately 17.7% lower MAE and 18.3% lower RMSE relative to ViT-S, and provides a standardized reference point for indoor object-level distance evaluation under the same-box protocol.

## 1. Introduction

Absolute distance estimation for individual objects from monocular images supports localization, interaction, and navigation in complex scenes. Related domains such as Dense monocular metric depth estimation [1,2,3,4,5] predicts per-pixel depth with scene-level metrics and monocular 3D object detection  [6,7,8,9,10] estimates volumetric representations with 3D overlap criteria, sometimes reporting centroid depth. Recently, a stream of per-object distance estimation methods combines detection with depth cues. Most of these methods were developed and tested for outdoor scenes, but outdoor benchmarks such as KITTI lack indoor counterparts. Furthermore, the use of different reference signals, aggregation rules, and differing fragmentation of datasets also makes cross-method comparison unreliable, and this is a reason to have a unified indoor evaluation protocol.

**Figure 1:**
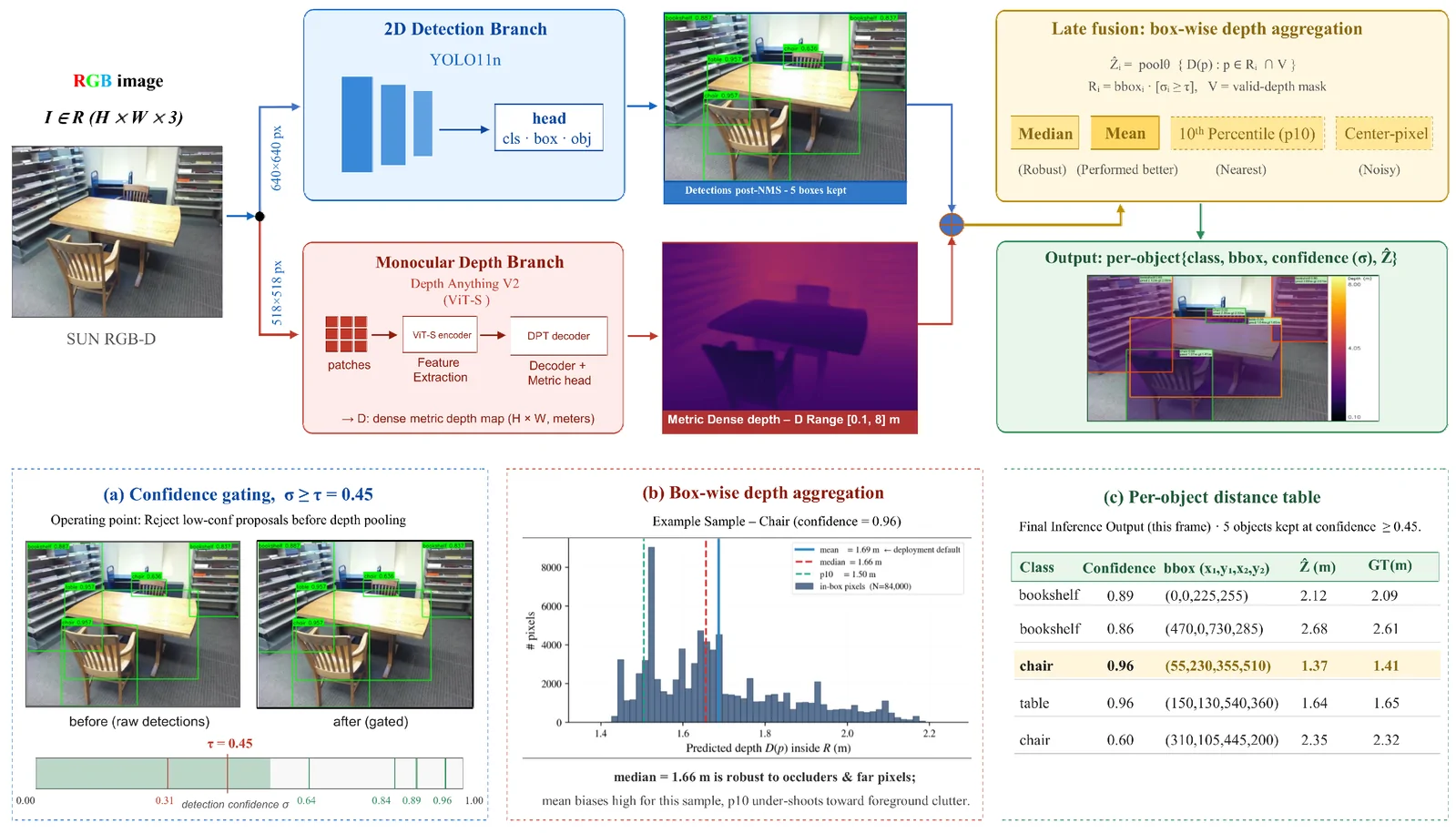
Overview of the late-fusion evaluation pipeline under the same-box protocol. A monocular RGB frame is processed independently by an object detector and a metric depth estimator. Each retained detection is matched to a ground-truth instance at the fixed operating point (conf = 0.45, IoU = 0.50), and depth values are aggregated within the same spatial region, i.e., the predicted box on both the predicted and ground-truth depth maps to yield one distance estimate per object. Detector and depth modules are trained independently. Distances shown are illustrative estimates from representative SUN RGB-D test frames. (**a**) shows the role of the confidence threshold in the selection of accurately detected objects for distance estimation. (**b**) shows the depth distribution within the predicted box of a sample object and the behavior of the aggregation methods. (**c**) lists per-object distances along with other details for the objects detected at the operating point. Evaluated aggregation strategies are mean, median, P10, and center-pixel.

In dense depth-based pipelines, object distance estimation combines localization, depth estimation, and region-wise aggregation, yet prior work differs in localization source, depth representation, and aggregation methods. Each of these choices changes reported performance even under conditions for the same models. We propose the same-box protocol (Figure 1) in which both predicted and reference distances are computed within the same predicted bounding box, standardizing the region of interest and removing dependence on ground-truth boxes at inference. Unlike GT segmentation masks [11], LiDAR centroids [12,13], or center-pixel sampling [14,15], the same-box protocol fixes the reference to the predicted box. We instantiate it with a modular detector–depth pipeline on SUN RGB-D. The contributions of this paper are as follows. First, we quantify the surface-to-centroid depth discrepancy on SUN RGB-D, establishing the magnitude of evaluation ambiguity between dense-depth and amodal 3D bounding-box distance representations (Section 5.3). Second, we propose the same-box evaluation protocol as a standardized, deployment-aligned methodology for per-object metric distance evaluation (Section 2). Third, we present a four-stage supervised training curriculum (Hypersim, ScanNet, NYU Depth V2, SUN RGB-D) achieving $\delta_{1}=0.928$ on NYU Depth V2 (Section 5.2). Fourth, we compare box-wise depth aggregation strategies under a shared operating point and report end-to-end indoor distance evaluation on the full SUN RGB-D test set, providing a reproducible reference for future work (Section 4.3). The remainder of the paper has been organized as follows: Section Related Work reviews methods related to the per-object distance estimation, Section 2 formalizes the same-box protocol, Section 2.3, Section 2.4, Section 3 and Section 4 describe the pipeline, setup, and results, Section 9 concludes.

### Related Work

Monocular depth estimation aims to infer a per-pixel depth map from a single RGB image. Recently, supervised monocular depth estimation has evolved from early convolutional regression architectures [16,17] to transformer-based foundation models. Encoder–decoder architectures with vision transformer backbones such as DPT [3] established the template for modern approaches. Adaptive bin decomposition methods, including AdaBins [18] and IEBins [19], improved metric accuracy by discretizing depth into learned intervals using a Swin-L backbone. Neural window CRF methods [20] further reduced error through structured prediction. Foundation models for depth estimation generalize more broadly across domains. For instance, Depth Anything V1 [21] showed that pretraining a DINOv2-initialized [22] encoder on 60 million unlabeled images yields strong zero-shot relative depth estimates. Depth Anything V2 [1] replaced noisy real-image pseudo labels with synthetic teacher supervision; the standard fine-tuned ViT-S variant achieves $\delta_{1}=0.961$ on NYU Depth V2. ZoeDepth [5] introduced relative-to-metric transfer through domain-specific metric-head fine-tuning. Metric3D v2 [23] reaches $\delta_{1}=0.989$ through canonical camera space normalization trained on 16 million images. UniDepth [4,24] and Depth Pro [2] extend zero-shot metric estimation to arbitrary cameras by conditioning on camera intrinsics.

An important observation that applies across all the methods reviewed above is that they are optimized and evaluated using per-pixel metrics computed over complete images. Their behavior when depth predictions are aggregated within detector-predicted bounding boxes, as required for per-object distance estimation, is not characterized by any existing benchmark. The shift from scene-level to object-level accuracy is a central concern of this paper.

The task of estimating a scalar metric distance to individual objects from a single image, using learning-based methods, was formally proposed by Zhu and Fang [12]. They trained an end-to-end network with region-of-interest pooled features and compared predictions against LiDAR centroid depths on KITTI [25] and nuScenes [26]. DistFormer [11] introduced masked object modeling pretraining and graph attention for per-object distance, reporting results on KITTI, nuScenes, and MOTSynth. In DistFormer, the reference depth is the median value within the ground-truth segmentation mask extracted from the ground-truth depth map, not within a predicted bounding box. DECADE [13] combines YOLOv8n detection with a distance regression network for vehicle collision avoidance and compares predictions against LiDAR range measurements on KITTI. All three methods target outdoor driving scenarios at distances of 5 to 150 m, address vehicle and pedestrian categories, and use LiDAR centroids or ground-truth segmentation masks as the reference signal.

Existing methods differ substantially in both scene domain and the reference signal used to define ground-truth distance. Zhu and Fang [12] and DECADE [13] evaluate against LiDAR-derived centroid distances for outdoor vehicles and pedestrians. DistFormer [11] uses a GT segmentation mask; the reference distance is the median depth inside the ground-truth mask region, a spatial support unavailable at deployment and disjoint from the predicted bounding box used during inference. These heterogeneous reference definitions make direct cross-method comparison unreliable, because improvements in reported metrics may reflect differences in evaluation protocol rather than estimation quality. The same-box protocol introduced here resolves this inconsistency; both the predicted and reference distances are extracted from the predicted bounding box, exactly matching what a deployed system computes. Table 1 compares these evaluation designs and summarizes evaluation diversity, highlighting the lack of a comparable benchmark within the indoor scenarios. Prior methods differ in domain, datasets, reference signal, and aggregation region, so cross-method ranking requires protocol alignment first. No prior work establishes a per-object distance evaluation framework on a standard indoor RGB-D benchmark using predicted bounding boxes and registered ground-truth depth maps under a protocol that is consistent with deployment behavior.

Many recent works combine an off-the-shelf monocular depth estimator with an object detector to produce per-object distances without end-to-end training. These late-fusion systems pass a single RGB image through both branches independently and extract a scalar distance from the predicted depth map inside each detected bounding box. The approach has been explored primarily in driving and outdoor settings. De Guzman et al. [28] pair MiDaS with YOLO and a lens optics calibration step. Thombre et al. [29] combine YOLOv8 with Depth Anything V1 and train a gradient boosting error correction model, reporting RMSE between 1.26 m and 1.58 m on Indian highway data. Cetin et al. [30] used YOLOv8s and Monodepth2 to introduce a weighted distance estimator that downweights boundary pixels. None of these systems address indoor benchmarks or report results under a standardized evaluation protocol. Late-fusion road and railway systems likewise combine detectors with monocular depth estimators [28,29,30], yet typically report application-specific errors without fixing confidence, IoU, matching, and reference-region conventions on a standard indoor RGB-D split; the same-box protocol is designed to make those conventions explicit before comparing models or aggregation rules. The choice of how to aggregate depth values within a bounding box varies across these systems. Center-point sampling is the simplest approach, adopted by CenterDepth [14] and YOLO MDE [15]. Region-based alternatives aggregate over valid pixels within the box. Masoumian et al. [31] compute the median of all depth map pixels inside each predicted bounding box. A recent benchmark on wildlife distance estimation [32] directly compares mean-based and median-based box extraction and reports that the median is more robust to extreme depth values. To our knowledge, no prior work reports a systematic aggregation-strategy comparison on SUN RGB-D under a shared operating point (conf = 0.45, IoU = 0.50) and one-to-one matching to valid ground-truth instances; Section 5.1 compares four evaluated strategies (mean, median, P10, and center-pixel) on matched true-positive pairs from the 5050-image test set.

## 2. Method

We propose a deployment-aligned framework for evaluating per-object metric distance estimation in indoor scenes. The framework is designed for dense depth-based late-fusion pipelines, where object distance is obtained by combining object detection with monocular depth estimation followed by region-wise aggregation.

### 2.1. Problem Formulation

Given an RGB image, the system produces a set of detected bounding boxes with associated confidence scores and a dense metric depth map. For each detected object, both the predicted distance and the reference distance are obtained by aggregating depth values within the same predicted bounding box, read from the predicted and sensor depth maps, respectively. Formally, let $B_{\mathrm{pred}}=\left\{b_{1},b_{2},\ldots ,b_{k}\right\}$ denote the predicted bounding boxes for a given image and let $D_{\mathrm{pred}}$ and $D_{\mathrm{gt}}$ denote the predicted and sensor depth maps. For each predicted box $b_{i}$,

(1) $${\hat{d}}_{i}=A\left(D_{\mathrm{pred}},b_{i}\right),\;d_{i}^{*}=A\left(D_{\mathrm{gt}},b_{i}\right),$$

where A() is an aggregation function that extracts a scalar distance from a depth map within a bounding box region. Valid depth pixels are those that are finite and fall within the range $\left[0.1,d_{\max}\right]$ m, where $d_{\max}=8$ m for SUN RGB-D evaluation. A predicted box is retained for evaluation only if it contains at least 64 valid depth pixels in $D_{\mathrm{gt}}$. Let $V\left(D,b_{i}\right)=\left\{\;D\left(p\right):p\in b_{i},\;D\left(p\right)\in \left[0.1,d_{\max}\right]\;\right\}$ denote valid depth values inside box $b_{i}$, with $\left|V\right|=n_{i}$ (see Algorithm 1). The aggregation functions are

(2) $$A_{\mathrm{mean}}=\frac{1}{n_{i}}\sum_{v\in V}v,\;A_{\mathrm{med}}=\mathrm{median}\left(V\right),\;A_{P10}=Q_{0.10}\left(V\right),$$

where $Q_{0.10}$ is the 10th-percentile of V. The center-pixel baseline reads $D(c_{i})$ at box center $c_{i}$. Mean aggregation is used for primary results unless stated otherwise; comparisons are discussed in Section 5.1. Algorithm 1 summarizes the object-wise distance aggregation procedure used at inference and evaluation. For box $b_{i}=\left[x_{1},y_{1},x_{2},y_{2}\right]$ and depth map *D*, valid pixels are

(3) $$V_{i}=\left\{\;D\left(u,v\right)|\left(u,v\right)\in b_{i},\;D\left(u,v\right)\;\mathrm{finite},\;d_{\min}\leq D\left(u,v\right)\leq d_{\max}\;\right\},$$

where $\left[d_{\min},d_{\max}\right]=\left[0.1,8.0\right]$ m on SUN RGB-D (consistent with Equation (1)). Boxes with valid pixels less than 64 ($|V_{i}|<64$) in Ground Truth Depth maps ($D_{\mathrm{gt}}$) are excluded from evaluation.
**Algorithm 1:** Object-wise metric distance aggregation from dense depth.
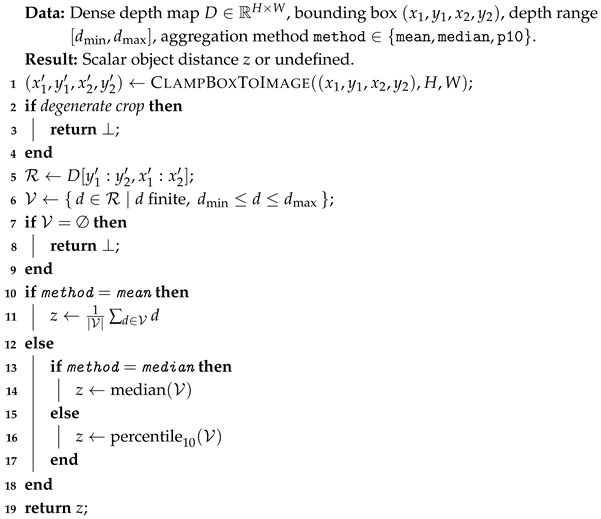


Reading both quantities from the same spatial region eliminates dependence on ground-truth bounding boxes or labels and aligns evaluation with what the deployed system computes at inference time. The ground-truth bounding box is used solely to establish object identity for matching, not to compute either distance. Throughout, $D_{\mathrm{gt}}$ denotes the official pre-processed SUN RGB-D depth map used as the reference signal.

#### Matching Criterion

Each predicted box is matched to the ground-truth box with the highest intersection over union, subject to a minimum IoU threshold $\tau_{\mathrm{IoU}}$. We adopt a lower threshold than the outdoor convention of 0.70 used by DistFormer [11] because indoor detection operates under heavier occlusion and clutter, and a stricter threshold would discard a large fraction of correctly localized objects. The primary evaluation uses $\tau_{\mathrm{IoU}}=0.50$ with a confidence threshold of 0.45, yielding 7876 matched true-positive pairs from a single fixed detection run at this operating point from the 5050-image SUN RGB-D test set. Matching is performed independently per image as a one-to-one greedy assignment. (i) Detections below the confidence threshold (conf<0.45) are not considered for final distance measurement. (ii) All IoU values between remaining predicted boxes and ground-truth boxes of the same canonical class are computed. (iii) Pairs are considered in descending IoU order; a pair $\left(\hat{b},b^{*}\right)$ is accepted if $\mathrm{IoU}\left(\hat{b},b^{*}\right)\geq \tau_{\mathrm{IoU}}=0.50$ and neither box is already matched. (iv) Each accepted pair contributes one matched detection. Remaining unmatched predictions are false positives and unmatched ground-truth boxes are missed detections, both excluded from distance metrics. Predictions are also excluded when no valid annotated instance exists for the matched class in the scene (e.g., in case of sparse GT annotations). This yields 7876 matched true-positive pairs on the 5050-image SUN RGB-D test set.

### 2.2. The Same-Box Evaluation Protocol

To ensure consistent per-object distance evaluation across different methods, we define a fixed region of interest, i.e., the predicted 2D bounding box. Unlike standard protocols that rely on heterogeneous region definitions (e.g., ground-truth boxes, segmentation masks, or heuristic crops), we compute both the predicted distance and the reference distance using the same region, the predicted box. Specifically, for each detected object, we extract depth values only from pixels falling inside its predicted 2D bounding box. The reference distance (e.g., from a dense depth map or LiDAR) is then computed over the identical set of pixels. This protocol mirrors deployment conditions, where ground-truth annotations are unavailable, removing heterogeneity in region definition while reflecting deployment conditions where only predicted boxes are available.

The protocol is intended for surface-based distance aggregation where both ${\hat{d}}_{i}$ and $d_{i}^{*}$ are scalars derived by aggregating depth values over the predicted 2D box (Equation (1)), consistent with dense-depth late-fusion pipelines. It does not directly produce centroid distances from amodal 3D boxes; extension to centroid-based evaluation would require an explicit geometric correction for the surface-to-centroid offset (median 0.414 m on SUN RGB-D; Section 5.3).

**Protocol Validation** To validate the same-box protocol and disentangle the contributions of detection error and depth estimation error, we compare three evaluation conditions on a fixed set of matched pairs. In the same-box condition, both the predicted depth map and the sensor depth map are read inside the predicted bounding box, so any observed error reflects depth estimation under the actual detector output. In the oracle-box condition, both maps are read inside the ground-truth bounding box, which replaces the detector with a perfect localizer and tests sensitivity to the choice of ground-truth region. In the localization-stress condition, the predicted depth map is read through the predicted box while the sensor depth map is read through the ground-truth box, which isolates the effect of spatial misalignment between the two regions. Section 4 presents the results and interpretation.

### 2.3. Late-Fusion Pipeline

To instantiate the framework, we use a domain-adapted object detector (YOLO11n) and a pretrained monocular metric depth estimator (Depth Anything V2 ViT-S). The detector provides 2D bounding boxes, while the depth model predicts per-pixel metric depth. These components are trained independently and are not modified for joint optimization.

The proposed system is a modular late-fusion pipeline in which a metric depth branch and an object detection branch process the same input image independently. The two branches are trained separately and no joint end-to-end training is performed. At inference time, the depth branch produces a dense metric depth map, the detection branch produces bounding boxes with confidence scores, and a lightweight aggregation module applies the box-wise depth aggregation defined in Section 2 to obtain a scalar distance estimate per detected object. Figure 1 illustrates the pipeline. Formally, $D=f_{\mathrm{depth}}\left(I\right)$, $\left\{\left(b_{i},c_{i}\right)\right\}=f_{\det}\left(I\right)$, and $z_{i}=g\left(D,b_{i}\right)$ with *g* given by Algorithm 1. Late fusion enables a modular design that has three practical advantages. It allows either branch to be replaced, retrained, or ablated independently, which facilitates the controlled experiments reported in Section 4. It enables each branch to use large-scale task-specific training data without requiring paired per-object distance annotations. It enables deployment flexibility, where faster detectors or more accurate depth models can be substituted without retraining the entire pipeline.

The depth branch is a Depth Anything V2 ViT-S model [1] with a DPT-style decoder and a metric depth head. Rather than single-stage fine-tuning on the target benchmark, the depth branch is trained through a four-stage supervised curriculum that progresses from large-scale synthetic geometric pretraining to domain-specific real indoor metric adaptation. The training sequence and role of each stage are summarized in Table 2.

Table 2 summarizes the four-stage curriculum: Hypersim synthetic pretraining, ScanNet real-domain bridge, NYU Depth V2 metric calibration, and SUN RGB-D target adaptation at 8 m. Stage-wise dense-depth and object-distance gains are reported in Section 4.

#### Loss Function and Optimization

The training objective in all depth training stages is to optimize the Scale-Invariant Logarithmic (SiLog) loss [16] with λ=0.5 on valid depth pixels:

(4) $$L_{\mathrm{SiLog}}=\sqrt{E\left[{\left(\log d-\log\hat{d}\right)}^{2}\right]-0.5\cdot {\left(E\left[\log d-\log\hat{d}\right]\right)}^{2}}$$

where *d* and $\hat{d}$ are ground-truth and predicted metric depth, respectively, and expectations are computed over valid depth pixels in each batch. The SiLog loss penalizes the scale-invariant logarithmic depth residual, while the λ=0.5 term reduces sensitivity to global scale offsets, which is important when training across datasets with different absolute depth distributions. Conservative handling of small valid-pixel counts prevents the scale term from becoming numerically unstable in sparse depth regions. The optimizer is AdamW [33] with weight decay 0.01 and momentum betas $\left(\beta_{1},\beta_{2}\right)=\left(0.9,0.999\right)$. A differential learning rate policy is applied with a base learning rate for pre-trained encoder layers, while the newly initialized parameters, such as the metric depth head, use a learning rate ten times larger.

This accelerates the adaptation of the task-specific components while preserving the geometric prior in the pre-trained encoder. Gradient clipping is applied to limit the gradient norm to 1.0. Standard data augmentation, including random horizontal flipping and optional color jitter, is applied throughout training.

### 2.4. Detection Branch

The detection branch uses YOLO11n [34], a single-stage detector with approximately 2.6 million parameters. The detector is fine-tuned on a YOLO-format representation of SUN RGB-D constructed through three preparation steps. First, a label mapping consolidates the 800-plus raw annotation strings into ten canonical indoor categories (chair, table, sofa, bed, desk, bookshelf, toilet, dresser, bathtub, night stand) matching the MMDet3D SUN RGB-D taxonomy. Second, inverse frequency class weighting and minority class oversampling address the severe imbalance between the most frequent categories (chair, 1708 instances) and the rarest (bathtub, 24 instances). Third, polygon annotations are converted to axis-aligned bounding boxes in YOLO format. Training uses AdamW with cosine annealing, automatic mixed precision, mosaic augmentation disabled for the final five epochs, and early stopping with a patience of 15 epochs (Table 3). At 640-pixel input resolution, the SUN-trained YOLO11n achieves mAP50 =0.878 on the validation split.

The ten-class vocabulary follows MMDet3D sunrgbd-3d-10 [36]. Chair accounts for ≈31% of training instances. Inverse-frequency class weighting and minority-class image oversampling mitigate imbalance. Annotations outside vocabulary are excluded by design, defining the system’s operating scope.

## 3. Experimental Setup

### 3.1. Datasets

Hypersim [37] provides photorealistic ray-traced RGB images paired with dense metric ground truth depth maps of diverse synthetic indoor scenes. The dataset contributes 59,543 training pairs and 7386 validation pairs. Depth values are converted to camera-ray depth before training. Hypersim’s role is limited to geometric warm-up pretraining. The geometric diversity of Hypersim across room types, furniture arrangements, and lighting conditions provides the depth encoder with a strong spatial prior that generalizes across real indoor distributions.

ScanNet [38] provides dense RGB-D sequences of real indoor scenes captured with a structured-light sensor. We use the official pre-processed subset of 25k images with corresponding depth maps. Our split uses 19,466 pairs for training and 5436 pairs for validation. ScanNet bridges the domain gap between Hypersim’s synthetic geometry and the real-image statistics of subsequent benchmark stages by exposing the model to realistic textures, illumination variations, and sensor noise patterns before metric calibration. ScanNet is used only for depth curriculum training and does not contribute to detector training or final test evaluation.

NYU Depth V2 [17] provides RGB-D frames of indoor scenes captured with a Microsoft Kinect v1 sensor. The standard Eigen split provides 43,245 training images, 4328 validation images, and 654 test images. All depth evaluations use the standard Eigen crop and a depth range of [0.1,10.0] m at an input resolution of 518×518.

SUN RGB-D [39] comprises 10,335 images collected across four sensor types (Kinect v1, Kinect v2, Intel RealSense, Asus Xtion). The leakage-safe split provides 4757 training, 528 validation, and 5050 test images. Ground-truth depth is taken from the official hole-filled depth maps.

Zero-shot benchmarks. DIODE indoor [40] (325 images) and DA-2K indoor [1] (m, 420 pairwise comparisons) are held out entirely from training and used to evaluate depth generalization without fine-tuning.

### 3.2. Evaluation Protocol and Metrics

Depth-only evaluation on NYU Depth V2 uses the standard protocol of Eigen et al. [16], with AbsRel, RMSE, $\log_{10}$, and threshold accuracy $\delta_{k}$ at k∈{1,2,3}. Per-object distance evaluation on SUN RGB-D uses MAE, RMSE, AbsRel, SqRel, and $\delta_{k}$ under the same-box protocol. For *N* matched objects with predicted distance ${\hat{d}}_{i}$ and reference $d_{i}^{*}$, $\mathrm{MAE}=\frac{1}{N}\sum_{i}\left|{\hat{d}}_{i}-d_{i}^{*}\right|$, $\mathrm{RMSE}=\sqrt{\frac{1}{N}\sum_{i}{\left({\hat{d}}_{i}-d_{i}^{*}\right)}^{2}}$, $\mathrm{AbsRel}=\frac{1}{N}\sum_{i}\left|{\hat{d}}_{i}-d_{i}^{*}\right|/d_{i}^{*}$, $\mathrm{SqRel}=\frac{1}{N}\sum_{i}{\left({\hat{d}}_{i}-d_{i}^{*}\right)}^{2}/d_{i}^{*}$, and $\delta_{k}$ is the fraction with $\max\left({\hat{d}}_{i}/d_{i}^{*},d_{i}^{*}/{\hat{d}}_{i}\right)<1.25^{k}$. Per-object distance metrics are computed on the SUN RGB-D test set (5050 images) by matching each predicted box to a valid ground-truth annotation at IoU ≥ 0.50 and confidence ≥ 0.45. The depth branch is trained on a multi-dataset curriculum (Hypersim, ScanNet, NYU Depth V2, SUN RGB-D), while the object detector is trained on the SUN RGB-D training split only. Detections without a corresponding annotated instance (e.g., missing GT annotations) are excluded from distance results but remain in detection precision and recall. Distance quality is reported conditionally on successful detection and end-to-end system coverage is summarized by detection recall, Table 13, and Figure 4. The primary operating point for all per-object distance results in this paper is $\tau_{\mathrm{IoU}}=0.50$, confidence threshold 0.45, depth cap 8 m for SUN RGB-D, canonical ten-class GT vocabulary, and official hole-filled ground truth. This configuration yields 7876 matched true-positive pairs from a single fixed detection run across the 5050-image test set and is applied uniformly throughout Section 4. Detection metrics (precision, recall, F1) are shown alongside per-class distance MAE in Figure 5.

### 3.3. Implementation Details

The depth branch is initialized from the official Depth Anything V2 ViT-S relative depth pretrained weights. Training uses AdamW with weight decay 0.01 and cosine learning rate annealing. All training uses 518×518 pixel input images and a batch size of 4 per GPU, distributed across 8 NVIDIA A40 GPUs. YOLO11n is trained at 640×640 pixels with a fixed random seed of 0 and early stopping with a patience of 15 epochs. Full pipeline throughput is measured by timing sequential depth inference, object detection, and distance aggregation over 50 inference iterations after 5 warm-up passes on a single NVIDIA A40 GPU. Each curriculum stage trains until validation $\delta_{1}$ plateaus (early stopping, patience 15), with the differential learning-rate policy above and cosine annealing per stage; batch size is 4 per GPU across 8 A40 GPUs at 518×518.

## 4. Results and Analysis

### 4.1. Depth Estimation Results on NYU and SUN RGB-D

#### 4.1.1. Metric Depth Evaluation on NYU Depth V2

The metric depth branch is first evaluated in isolation on the standard NYU Depth V2 Eigen test split (654 images, Eigen crop [45:471,41:601], depth range [0.1,10.0] m, input resolution 518×518) prior to downstream object-level evaluation. This experiment verifies that the proposed curriculum produces a competitive indoor metric depth model, and identifies suitable checkpoints for integration into the per-object distance estimation pipeline.

Table 4 compares the curriculum-trained Depth Anything V2 variants against representative supervised monocular depth estimation methods on NYU Depth V2. Recent depth estimation models already achieve strong performance on dense indoor benchmarks, including transformer-based approaches such as NeWCRFs [20], UniDepth V2 [4], Metric3D [23], and Depth Pro [2]. Consequently, the objective of this work is to study how metric depth predictors can be adapted and evaluated for downstream per-object distance estimation under a controlled indoor evaluation protocol, rather than to propose a new state-of-the-art dense-depth architecture.

The curriculum-trained ViT-S model reaches $\delta_{1}=0.9286$ and AbsRel =0.0922; ViT-L improves to $\delta_{1}=0.9493$ (Table 4). A gap to official Depth Anything V2 NYU numbers is expected because our pipeline starts from the Hypersim metric checkpoint and uses indoor-only supervision without teacher distillation. Our goal is domain adaptation for object-level evaluation, not dense-depth leaderboard performance. ViT-S remains the preferred deployment backbone in the integrated pipeline.

#### 4.1.2. Metric Depth Evaluation on SUN RGB-D

The depth branch is further evaluated on SUN RGB-D to assess the effect of progressive indoor curriculum training and target-domain adaptation. Table 5 reports isolated dense-depth performance for ViT-S and ViT-L backbones under different training conditions.

The curriculum-trained models achieve lower error than reduced-training baselines. For ViT-S, the full four-stage curriculum (Hypersim → ScanNet → NYU Depth V2 → SUN RGB-D) achieves $\delta_{1}=0.881$, improving over the SUN RGB-D-only baseline ($\delta_{1}=0.839$) while reducing AbsRel from 0.318 to 0.149 and RMSE from 1.110 m to 0.370 m. Large-scale pretraining on synthetic and intermediate indoor datasets therefore provides geometric priors that single-dataset training does not recover. A similar trend appears for the ViT-L backbone. The checkpoint after Stage 3 (Hypersim → ScanNet → NYU Depth V2) yields $\delta_{1}=0.859$; SUN RGB-D adaptation raises this to $\delta_{1}=0.916$ and further reduces AbsRel and RMSE. The final SUN RGB-D fine-tuning stage improves domain-specific metric calibration despite the relatively small training split.

Overall, the results show that progressive indoor curriculum training and target-domain adaptation both contribute substantially to dense-depth quality on SUN RGB-D and downstream per-object distance estimation. Although SUN RGB-D serves primarily as the benchmark for downstream per-object distance evaluation in this work, the isolated dense-depth analysis confirms that the depth branch maintains strong metric consistency prior to integration with the object detection pipeline.

### 4.2. Comparison of the SOTA Metric Depth Estimation Models for Per-Object Distance Estimation

This section evaluates several recent pre-trained monocular metric depth estimation models under the proposed same-box object-distance protocol on SUN RGB-D. The comparison includes UniDepth2, Metric3D v2, DepthPro, and Depth Anything V2 (DA2 Hypersim) using all publicly available encoder variants. For all experiments, YOLO11n detections, confidence thresholds, IoU thresholds, and same-box reference depths are fixed; only the depth predictor and box-wise aggregation statistic are varied. Object distances are computed from hole-filled SUN RGB-D depth maps using mean, median, and 10th-percentile (P10) aggregation within each predicted bounding box. Unlike conventional dense-depth evaluation, the objective here is object-level metric distance quality rather than full-image reconstruction accuracy. The experiments evaluate how well pretrained depth representations transfer to object-centric geometric reasoning without additional indoor adaptation. All models in this section are publicly released pretrained metric depth estimators, evaluated without training, fine-tuning, or indoor adaptation (Table 6, Table 7 and Table 8).

#### 4.2.1. Mean Pooling

Table 6 reports results using arithmetic mean pooling within each detected object region. Metric3D v2 achieves the strongest overall performance, with the ViT-S encoder obtaining the lowest MAE (0.411 m), lowest RMSE (0.479 m), and highest $\delta_{1}$ (0.656). DepthPro performs competitively relative to UniDepth2 despite using a single pretrained configuration, indicating comparatively strong zero-shot metric calibration on indoor scenes.

UniDepth2 achieves intermediate performance across all encoder variants, with relatively consistent metrics between ViT-S, ViT-B, and ViT-L. In contrast, the DA2 Hypersim checkpoints perform substantially worse under the same evaluation setting, with MAE values approaching 0.9 m and low threshold accuracy. This pattern reflects a substantial domain gap between synthetic metric pretraining and real indoor RGB-D evaluation when no additional adaptation is applied. A notable observation across all model families is that larger encoders do not consistently improve object-level distance estimation. In several cases, ViT-S outperforms ViT-B and ViT-L under the same protocol. This suggests that improvements in dense-depth representation capacity do not necessarily translate into improved object-level metric aggregation performance.

#### 4.2.2. Median Pooling

Table 7 presents the same evaluation using median pooling. The overall ranking of methods remains largely unchanged, with Metric3D v2 again achieving the strongest performance. Median aggregation slightly improves robustness for several models by reducing the influence of outlier depth pixels near object boundaries and partially occluded regions.

DepthPro and UniDepth2 also improve modestly relative to mean pooling, particularly in MAE and RMSE. The DA2 Hypersim models remain much weaker than the other pretrained methods, although median aggregation reduces error slightly compared to mean pooling. Better aggregation alone cannot close the underlying synthetic-to-real calibration gap. Across methods, the difference between mean and median pooling is relatively small compared to the performance gap between model families. This suggests that the quality of the underlying depth representation has a larger effect on object-level distance estimation than the choice of aggregation statistic alone.

#### 4.2.3. 10th Percentile (P10) Pooling

Table 8 evaluates 10th-percentile pooling, which weights near-surface depth responses within the detected object region. P10 pooling consistently reduces MAE across nearly all methods, with Metric3D v2 ViT-S achieving the lowest MAE among compared zero-shot models (0.365 m).

However, this reduction in absolute distance error is accompanied by substantially worse relative metrics and threshold accuracy. For example, $\delta_{1}$ decreases across all models compared to mean and median pooling. This behavior indicates that lower-percentile aggregation biases predictions toward foreground surface pixels, improving near-surface localization while simultaneously introducing systematic underestimation for thicker or volumetric objects. The trade-off observed with P10 pooling is particularly important for indoor scenes containing furniture, cabinets, and cluttered objects with significant depth extent. Although lower-percentile pooling can improve near-surface localization accuracy, it produces less stable global metric estimates than mean aggregation under the same evaluation protocol. Across aggregation rules, Metric3D v2 shows the strongest zero-shot object-distance transfer; DA2 Hypersim lags without indoor adaptation (Table 6, Table 7 and Table 8). Pixel-wise dense-depth accuracy alone does not predict object-level performance under the same-box protocol.

Overall, the results demonstrate that pretrained monocular depth models cannot be assumed to transfer uniformly to downstream object-level distance estimation tasks. The proposed same-box protocol exposes substantial differences between dense-depth quality and object-centric metric reliability, motivating the need for evaluation methodologies specifically designed for per-object distance estimation in indoor scenes.

### 4.3. Primary Per-Object Distance Estimation Results

The primary evaluation of the proposed system measures object-wise metric distance estimation under the deployment-aligned same-box protocol introduced in Section 2.2. The complete pipeline combines the SUN RGB-D-trained YOLO detector with the curriculum-trained monocular metric depth branch, and evaluates object distances on the official 5050-image SUN RGB-D test split using hole-filled depth maps as reference supervision. Distance estimates are computed inside each matched detection bounding box using mean, median, and 10th-percentile (P10) aggregation.

The evaluation is designed to assess whether monocular metric depth models, originally optimized for dense pixel-wise estimation, can provide reliable object-level metric distances in realistic indoor scenes. Unlike standard dense-depth benchmarks, the present protocol evaluates depth only within detected object regions, making the task sensitive to detector localization noise, object boundaries, occlusions, and foreground-background mixing. Table 9 and Table 10 report the complete integrated evaluation for curriculum checkpoints saved after Stage 4 (SUN RGB-D adaptation) and Stage 3 (NYU adaptation), respectively. All evaluations share the same fixed detection run (n=7876 matched true-positive pairs), matching protocol ($\tau_{\mathrm{IoU}}=0.50$, conf =0.45), and aggregation rules, isolating the contribution of the final SUN RGB-D adaptation stage to downstream object-wise distance estimation. Table 9 lists frozen-integrity Stage 4 metrics (ViT-S mean: MAE 0.1286 m, RMSE 0.1817 m, $\delta_{1}$ 0.9785; ViT-L mean: MAE 0.1059 m; all rows n=7876).

A consistent performance gain is observed after the final SUN RGB-D adaptation stage across all Depth Anything V2 variants. For the ViT-L backbone, MAE improves from 0.1732 m at the NYU stage to 0.1059 m after SUN RGB-D adaptation under mean aggregation, corresponding to an approximately 38.6% reduction in absolute distance error. Similar gains are observed for ViT-B (0.1676 m → 0.1087 m) and ViT-S (0.1851 m → 0.1286 m). Improvements are similarly reflected in RMSE, AbsRel, and $\delta_{1}$, indicating that the final curriculum stage improves both metric calibration and robustness across object instances.

These gains show that dense-depth performance on NYU Depth V2 does not transfer directly to object-wise distance estimation on SUN RGB-D without target-domain adaptation. Stage 3 already exposes the model to real indoor sensor statistics through ScanNet and NYU Depth V2, but the final SUN RGB-D stage further adapts the depth branch to the scene composition, annotation characteristics, and object distributions used at evaluation. The staged curriculum design is therefore validated: Hypersim synthetic supervision establishes global metric structure, ScanNet and NYU reduce the synthetic-to-real gap, and SUN RGB-D fine-tuning optimizes the model for object-centric evaluation.

Among the evaluated Depth Anything V2 backbones, ViT-L achieves the strongest overall performance after SUN RGB-D adaptation, reaching MAE = 0.1059 m, RMSE = 0.1484 m, AbsRel = 0.0553, and $\delta_{1}=0.9898$ under mean aggregation. ViT-B performs comparably, with only marginally higher error, while offering substantially lower computational cost. ViT-S remains competitive despite its smaller capacity, achieving MAE = 0.1286 m and $\delta_{1}=0.9785$. The relatively small performance gap between ViT-B and ViT-L suggests diminishing returns from increasing encoder scale once the model has been sufficiently adapted to the target domain. In contrast, the improvement from ViT-S to ViT-B is more substantial, indicating that moderate increases in representational capacity benefit object-level metric calibration more consistently than scaling to very large backbones.

Mean aggregation produces the strongest overall performance across nearly all metrics after SUN RGB-D adaptation. For ViT-L, mean aggregation achieves lower RMSE and higher $\delta_{1}$ than both median and P10 pooling, while maintaining the lowest AbsRel. Similar behavior is observed across ViT-S and ViT-B. The advantage of mean aggregation in the present evaluation differs from earlier experiments on raw SUN RGB-D depth maps, where median pooling was more stable. Hole-filled depth supervision reduces sparsity and invalid-pixel artifacts within object regions, making depth distributions inside boxes more spatially uniform. Under these conditions, averaging all valid pixels is beneficial because the estimator can use a denser, less corrupted geometric signal. Median aggregation still tolerates outliers and boundary contamination, but its advantage shrinks once missing-depth regions are corrected through hole filling.

The P10 estimator consistently produces the lowest MAE in several settings, particularly for ViT-B and ViT-L, indicating that near-surface pixels often better approximate the physically closest visible object surface. However, P10 simultaneously increases AbsRel and reduces $\delta_{1}$, reflecting reduced stability and greater sensitivity to local noise. This behavior is expected because lower-tail statistics favor boundary and foreground pixels, which can fluctuate under small depth perturbations or imperfect box alignment. Consequently, P10 acts as an aggressive near-surface estimator rather than a globally stable object-distance statistic. Overall, mean aggregation provides a favorable balance between absolute accuracy, relative calibration, and threshold consistency on hole-filled SUN RGB-D depth maps, and is therefore adopted as the primary deployment configuration.

Depth Pro exhibits a substantially different adaptation profile from Depth Anything V2. After SUN RGB-D adaptation, Depth Pro achieves competitive object-level performance, reaching MAE = 0.1615 m and $\delta_{1}=0.9718$ under mean aggregation. However, its Stage 3 NYU checkpoint performs considerably worse, with MAE exceeding 0.48 m across all aggregation strategies. This large performance gap indicates that Depth Pro requires stronger target-domain adaptation for reliable object-wise metric calibration in indoor scenes. Unlike the curriculum-trained Depth Anything variants, the pretrained Depth Pro checkpoint does not transfer effectively to the SUN RGB-D object-centric evaluation setting without explicit adaptation to the target domain distribution. Nevertheless, after SUN RGB-D fine-tuning, the model improves substantially, confirming that the downstream performance limitation originates primarily from domain mismatch rather than architectural incapacity.

After SUN RGB-D adaptation, ViT-L reaches about 11 cm mean object distance error with $\delta_{1}$ near 0.99 (Table 9). Strong dense-depth models still need target-domain adaptation and a fixed aggregation rule before object-level metrics stabilize. More broadly, the results show that strong dense-depth models do not automatically yield strong object-wise distance estimates in downstream settings. The final adaptation stage, aggregation strategy, and domain alignment all significantly influence downstream metric performance. The proposed curriculum therefore serves not as a novel depth architecture, but as a systematic adaptation framework that transforms high-capacity monocular depth estimators into reliable object-centric metric perception models for indoor scene understanding.

### 4.4. Qualitative Results

**Figure 2:**
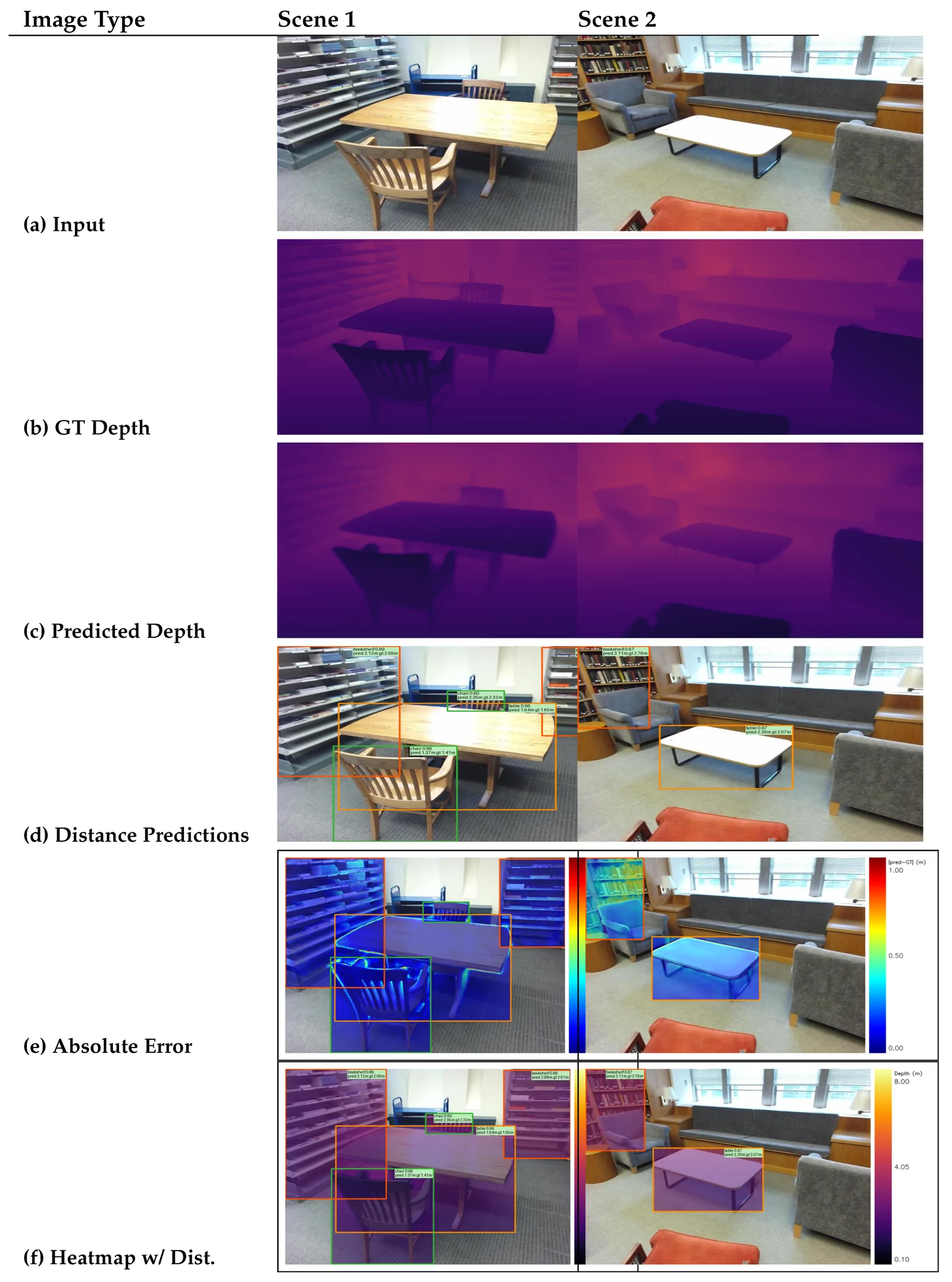
Qualitative depth and distance estimation results on SUN RGB-D test scenes (two scenes shown). Our domain-adapted Depth Anything V2 model produces metric depth maps (**c**) that accurately predict metric depth. (**a**,**b**) show the input images and ground truth depth maps, respectively. The proposed late-fusion pipeline predicts per-object distances (**d**,**f**) with minimal residual error (**e**). Errors primarily concentrate at object boundaries.

**Figure A2:**
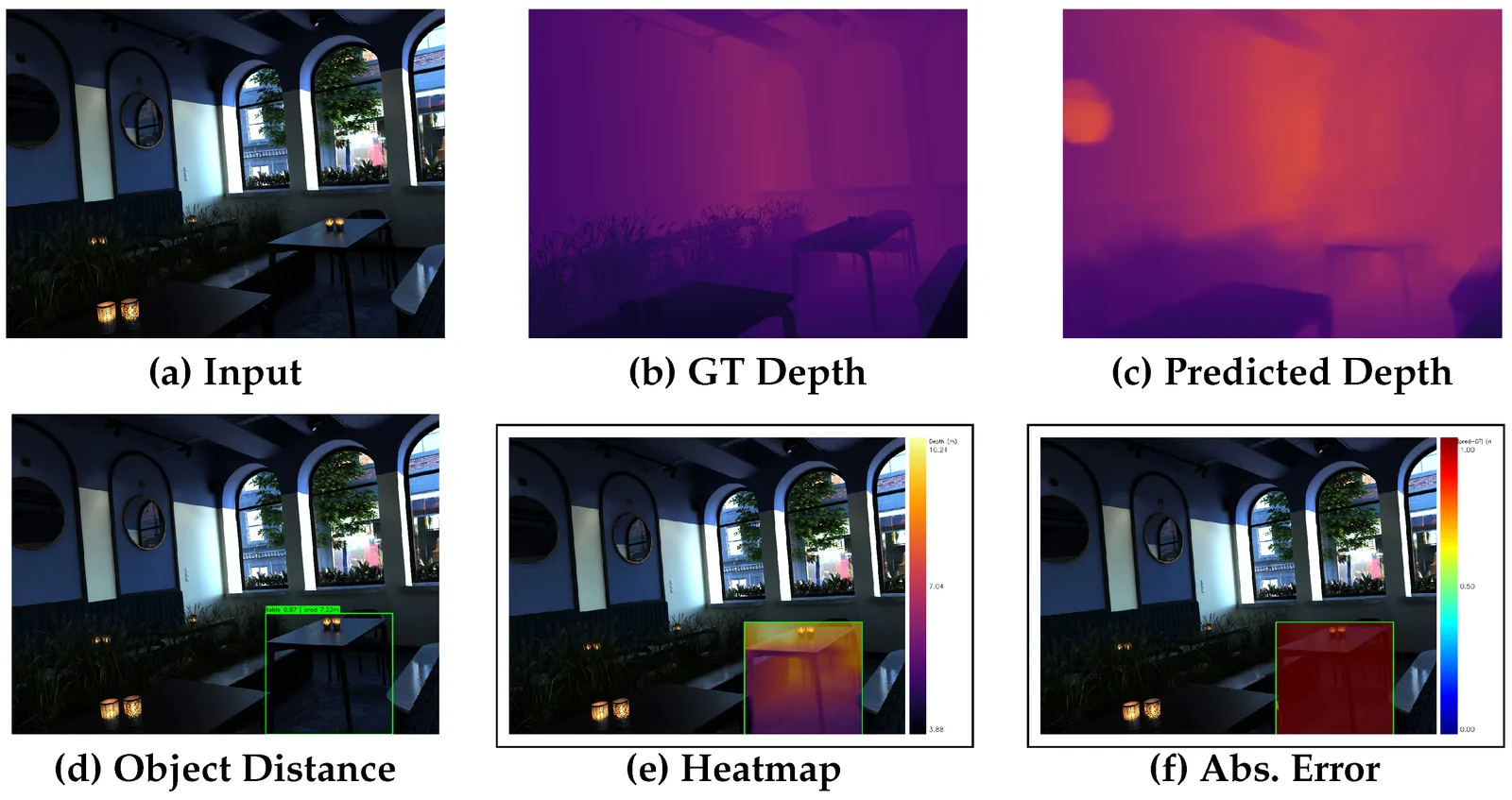
Cross-dataset qualitative result on ScanNet (zero-shot relative to SUN RGB-D training).

To better analyze the qualitative performance, we visualize the input and output of our method, as shown in Figure 2. The Figure shows two SUN RGB-D test scenes arranged as rows (a)–(f). Predicted depth (c) preserves ground-truth ordering (b), and per-object distances appear on detected boxes in rows (d)–(f). Residual error concentrates near boundaries and thin structures, consistent with the quantitative analysis. An additional ScanNet cross-dataset example is in Appendix Figure A2.

### 4.5. Detector Evaluation and Integrated Distance Estimation

**Figure 3:**
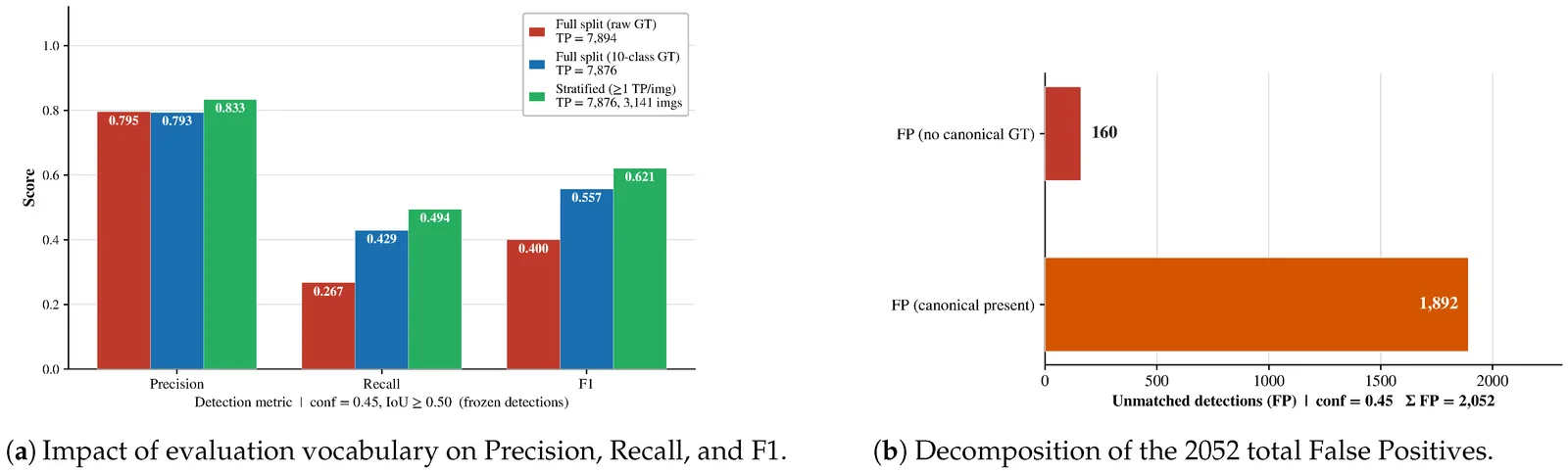
Detection metrics across evaluation scopes. (**a**) Evaluating against the full raw SUN RGB-D labels artificially deflates detection metrics due to 11,215 out-of-vocabulary objects. While precision is nearly unchanged between raw and canonical scopes, recall and F1 rise once OOV GT labels are excluded. Therefore, restricting evaluation to the 10 canonical training classes results in a fair assessment (P=0.793,R=0.429). (**b**) False Positive analysis reveals that only 7.8% of FPs are hallucinated in images with OOV objects. The remaining 92.2% occur in scenes with valid canonical objects, primarily due to confidence scores below the 0.45 threshold and spatial misalignment (IoU < 0.5). Only a small fraction of FPs are due to annotation sparsity or incorrect class prediction.

**Figure 4:**
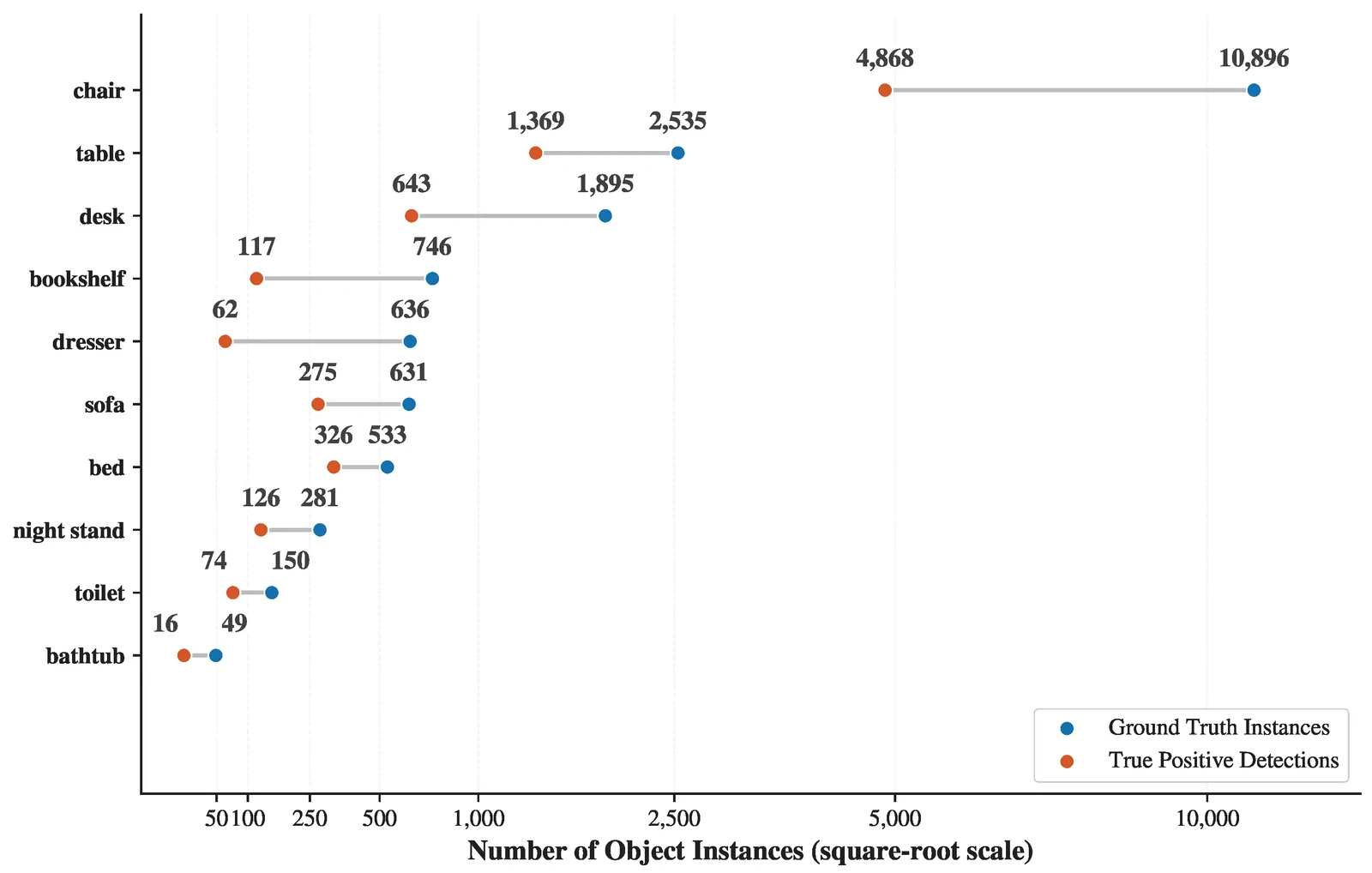
Per-class coverage on the 5050-image SUN RGB-D test set (conf = 0.45, IoU = 0.50): for each of the ten display-name classes, dumbbell markers show canonical GT count and matched true positives (TP), connected by the false-negative gap (FN = GT−TP). Aggregate matched pairs n=7876 from a single fixed detection run; the full canonical mapped total is 18,352 including alias raw labels. Unmatched false positives and missed detections contribute to precision and recall but are excluded from distance metrics.

The YOLO11n detector is evaluated on the official SUN RGB-D test split (5050 images) at the fixed operating point used throughout this paper (conf=0.45, IoU=0.50). SUN RGB-D annotates more than 800 raw labels, whereas the detector is trained on ten canonical indoor categories. Table 11 summarizes the GT vocabulary composition and image-level statistics on the SUN RGB-D test split. At the deployment operating point on canonical-class ground truth, the detector object counts are reported in Table 12 and it achieves precision =0.793, recall =0.429, and F1 =0.557 (Table 13 and Figure 3). Precision is favored over recall because false positives with failing IoU threshold attach distance estimates to invalid spatial regions, whereas missed detections only reduce coverage. To ensure reproducible cross-model comparison, all distance results use detections fixed from a single inference run at the operating point (confidence 0.45, IoU 0.50), yielding 7876 matched true-positive pairs (Figure 4) under greedy one-to-one class-aware matching. Distance MAE and RMSE are computed only on these pairs; unmatched predictions and missed ground-truth objects are included in precision and recall.

**Figure 5:**
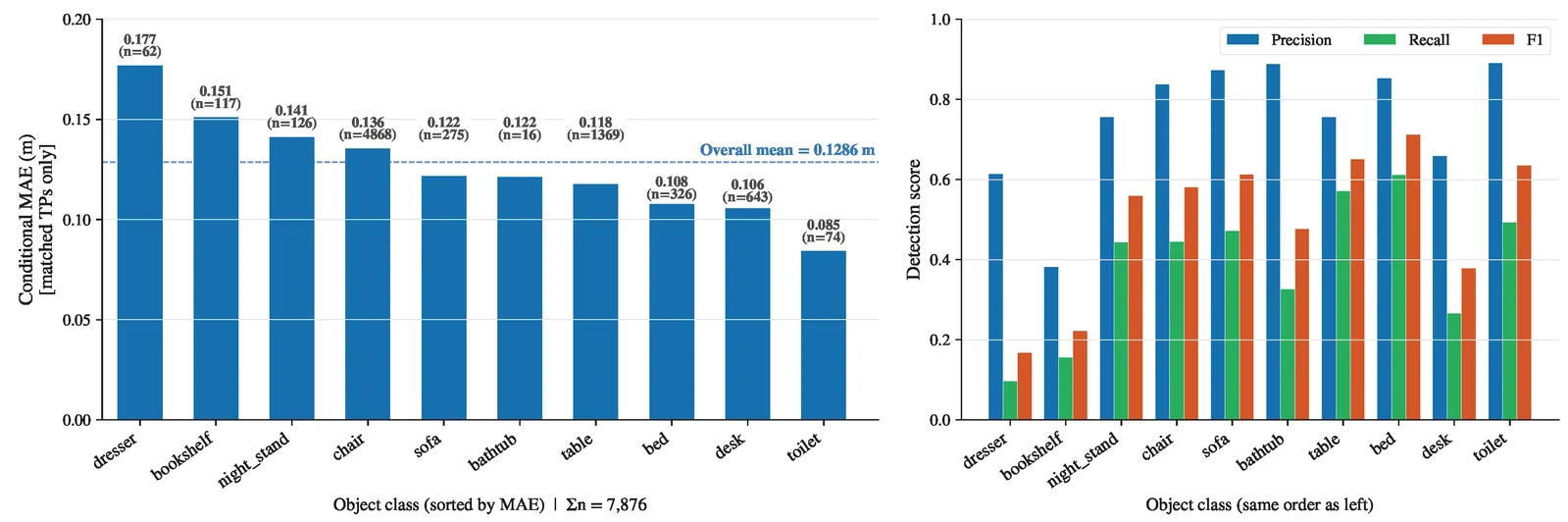
Per-class distance MAE (mean aggregation, same-box protocol) alongside per-class detection precision, recall, and F1 for the 10 canonical SUN RGB-D classes on 7876 matched pairs. Rare classes with low precision or recall tend to exhibit higher distance MAE.

**Figure A4:**
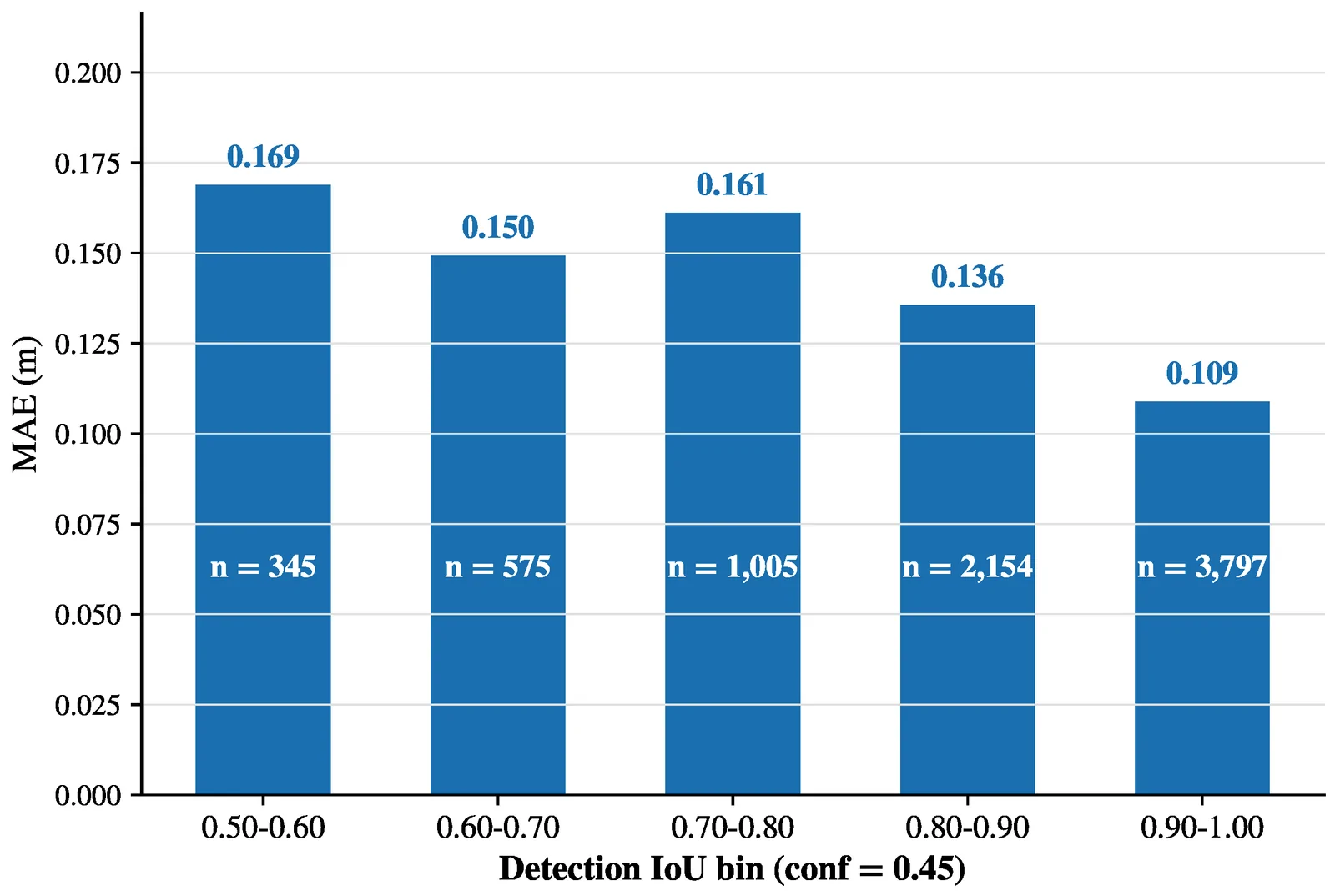
Impact of IoU on per-object distance MAE under same-box evaluation. The figure shows MAE by detection IoU bin at conf = 0.45, with sample counts per bin (n=345 to n=3797). MAE decreases from 0.169 m (IoU 0.50–0.60) to 0.109 m (IoU 0.90–1.00).

**Figure A5:**
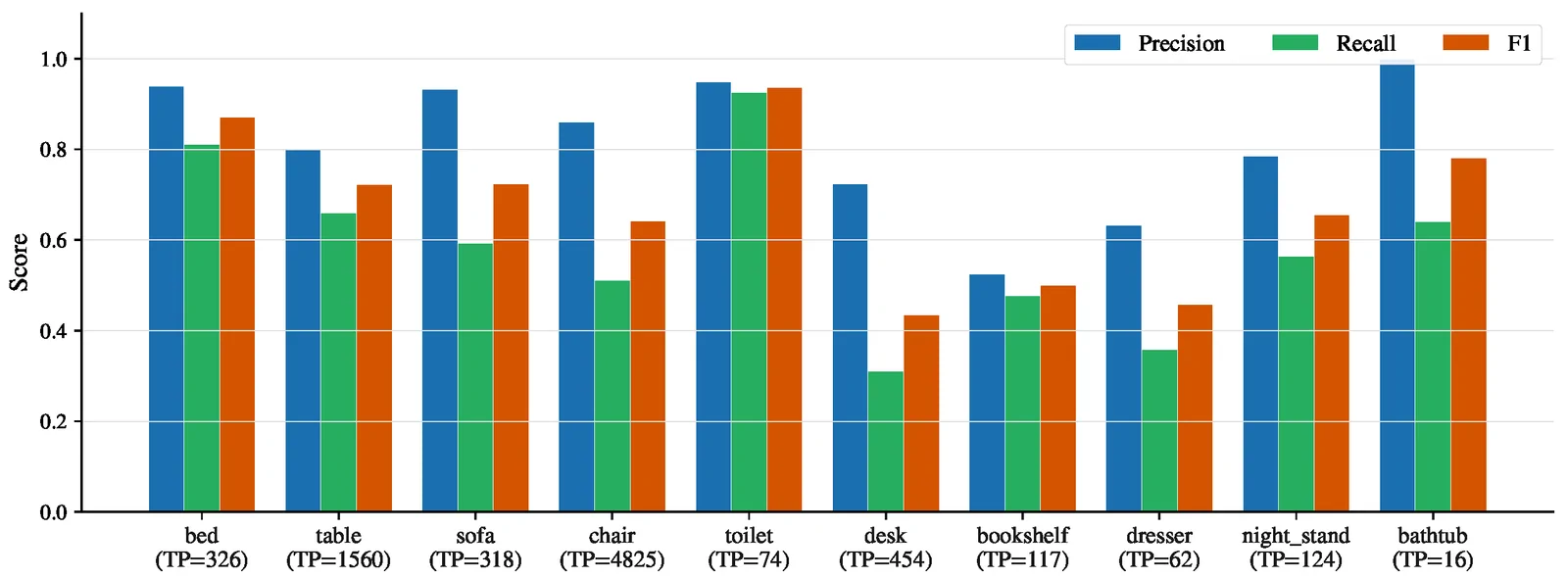
Grouped bars show Precision, Recall, and F1 for the 10-class canonical vocabulary with 7876 matched pairs at operating point conf = 0.45 and IoU = 0.50.

Distance quality is reported conditionally on successful detection. MAE characterizes depth-and-aggregation error given a correct localization, whereas recall of 0.429 summarizes end-to-end coverage over canonical-class objects. Figure 4 compares per-class ground-truth counts with matched pairs; Figure 5 integrates per-class detection scores with conditional distance MAE. High-recall, geometrically distinctive classes (bed, table, sofa, toilet) combine strong detection with lower MAE, whereas rare or ambiguous categories (dresser, bookshelf, bathtub) contribute disproportionate residual error. Count-weighted and recall-weighted mean per-class MAE (0.1287 m and 0.1286 m, respectively) indicate that low-recall classes do not inflate the aggregate metric. Confidence-threshold sensitivity is presented in Appendix Figure A4, per-class detection detail in Appendix Figure A5. Validation-split mAP@50 =0.878 and mAP@50–95 =0.743 reflect training-level performance and test-set precision/recall/F1 at conf =0.45 given in Table 13.

Table 14 evaluates the sensitivity of object-distance estimation to the choice of spatial support under three controlled conditions on the same 7876 matched pairs. All conditions share the same Depth Anything V2 ViT-S depth model, YOLO11n detection pipeline (conf = 0.45, IoU = 0.50), mean aggregation, official hole-filled SUN RGB-D ground-truth depth, and canonical ten classes; only the spatial support for reading predicted and reference depths changes. Under the deployment-aligned same-box condition, both maps are aggregated inside the predicted box $\left({\hat{B}}_{i},{\hat{B}}_{i}\right)$, yielding MAE = 0.1286 m, AbsRel = 0.0662, and $\delta_{1}=0.9785$. Under the oracle-box condition, both maps are aggregated inside the ground-truth annotation box $\left(B_{i}^{*},B_{i}^{*}\right)$, yielding MAE = 0.1289 m (difference 0.0003 m). The near-identical same-box and oracle-box scores indicate that, for correctly localized detections (IoU > 0.5), the predicted bounding box provides a spatial support that is statistically equivalent to the ground-truth annotation in terms of depth distribution. Therefore, swapping the predicted box for the annotated box yields a negligible change in aggregated distance. Although this does not indicate robustness to localization mismatch. The localization-stress condition $\left({\hat{B}}_{i},B_{i}^{*}\right)$ isolates this exact effect (Table 14). The resulting MAE of 0.1379 m (7.2% higher than same-box) demonstrates that introducing inconsistent spatial support, even while holding the depth model fixed, introduces measurable evaluation bias. These results support two observations. First, same-box and oracle-box isolate depth-and-aggregation error under a shared spatial support and therefore cannot diagnose localization mismatch; that effect is measured by localization stress. Second, maintaining identical spatial support for prediction and reference aggregation is important for stable and reproducible object-level distance evaluation.

**Figure A6:**
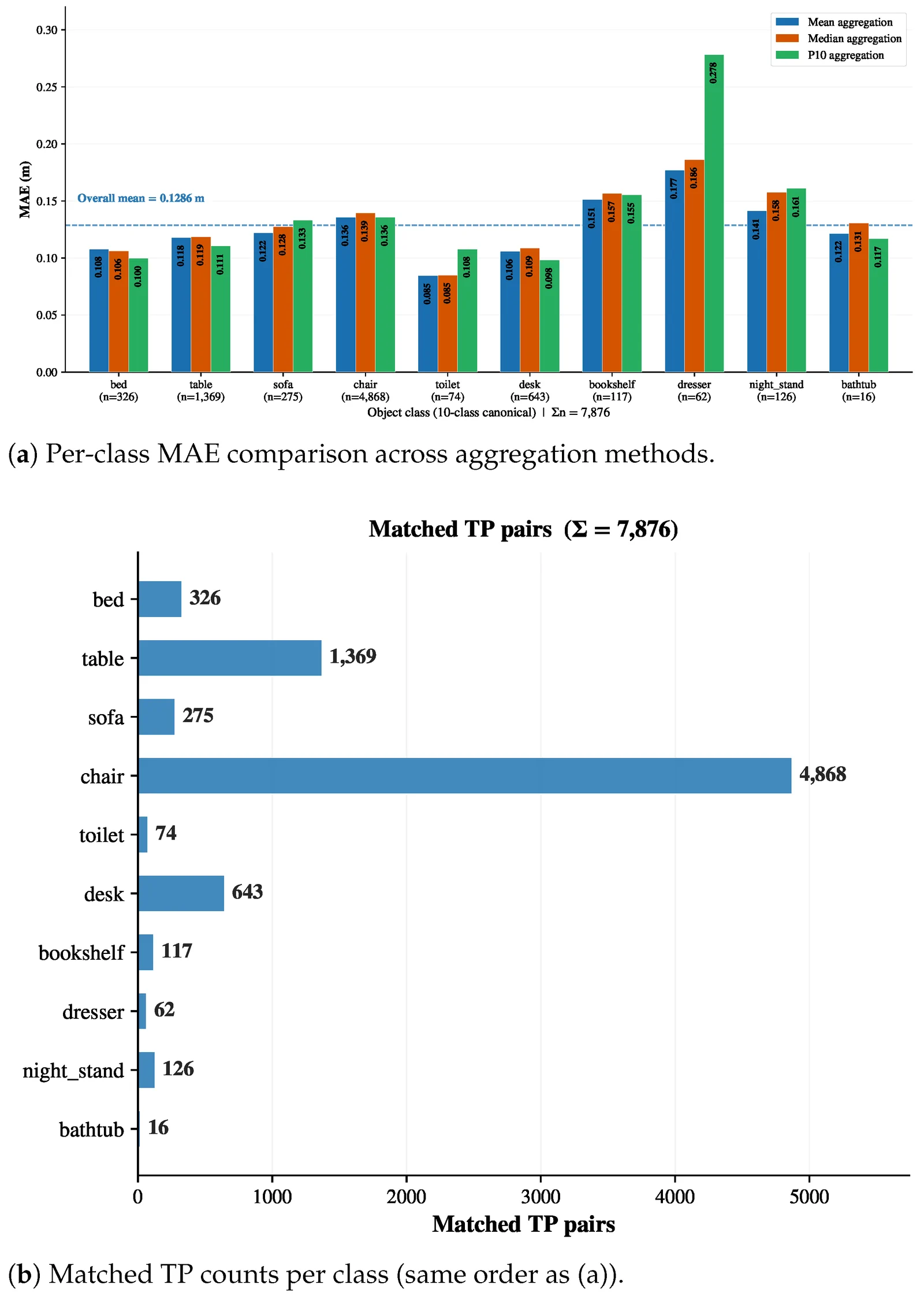
Per-class performance and data distribution for matched depth pairs. (**a**) Grouped bar chart comparing Mean, Median, and P10 aggregation for the 10 canonical classes. The dashed line indicates the global MAE of 0.1286 m (n=7876). Mean aggregation is consistently the most stable. (**b**) Distribution of matched TP counts for the same class order. The dataset is highly imbalanced: chair dominates with n=4868 pairs (61.8% of total). Sparse classes such as bathtub (n=16) and dresser (n=62) are more susceptible to aggregation fluctuations.

Per-class detection metrics are summarized in Appendix Figure A5 and Figure 5 integrates detection with distance MAE. Class-dependent detection quality propagates into distance error, while the primary operating point remains sufficient for same-box evaluation. High-frequency, geometrically distinctive classes (bed, table, sofa) combine strong detection (recall > 0.75) with the lowest distance MAE (<0.13 m). Rare or visually ambiguous categories drive the remaining error: dresser (n=59 matched pairs) and night_stand show moderate recall but elevated MAE (∼0.15–0.18 m), while bathtub (n=16) combines very low precision with higher MAE variability. Count-weighted mean per-class MAE is 0.1287 m; recall-weighting yields 0.1286 m. Per-class mean versus median aggregation is reported in Appendix Figure A6a. Confidence- and IoU-dependent distance sensitivity under the frozen operating point is reported in Appendix Figure A4. Protocol-level same-box, oracle-box, and localization-stress definitions follow Table 14.

### 4.6. Zero-Shot Generalization: DIODE Indoor and DA-2K

Zero-shot depth generalization on held-out DIODE indoor and DA-2K benchmarks is reported in Appendix Table A2.

## 5. Ablation Studies

### 5.1. Aggregation Strategy Ablation

**Figure 6:**
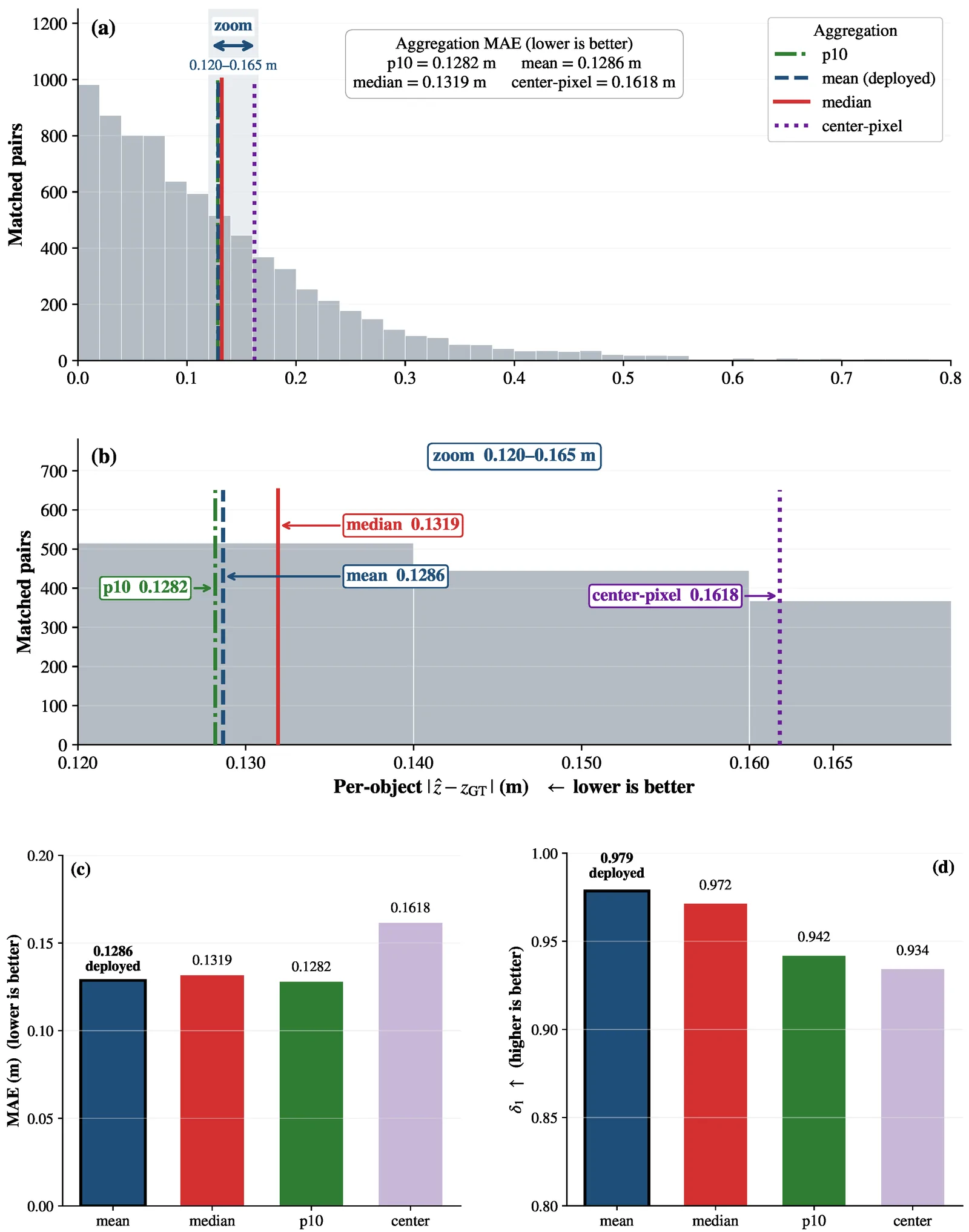
Evaluation of bounding-box aggregation strategies for per-object distance estimation. (**a**) shows the distribution of per-object absolute depth error, (**b**) comparing aggregation lines in the 0.12–0.165 m range. (**c**) Dataset MAE (↓ lower is better). (**d**) Dataset $\delta_{1}$ accuracy (↑ higher is better). We adopt the Arithmetic Mean as our deployed method (indicated by the thick black border in (**a**)), as it provides the most statistically robust balance of lowest RMSE, AbsRel, and SqRel, alongside the highest $\delta_{1}$ accuracy. (Operating point: conf = 0.45, IoU = 0.50, N = 7876).

We evaluate four box-wise depth aggregation strategies on the same 7876 matched SUN RGB-D pairs (Table 15 and Figure 6) i.e., arithmetic mean, median, 10th-percentile (P10), and center-pixel sampling. The ablation yields three consistent observations across backbone architectures and training stages.

First, region-based pooling (mean, median, P10) substantially outperforms center-pixel sampling (0.1618 m MAE versus 0.106–0.132 m under the primary operating point), confirming that aggregation over the full predicted box is necessary under real detector outputs.

Second, the arithmetic mean achieves the best or tied-best $\delta_{1}$ and AbsRel across Stage 4 backbones (e.g., ViT-S $\delta_{1}=0.9785$ vs. median 0.9716 and P10 0.9421), reversing the usual median preference on raw sensor depth because hole-filled supervision reduces in-box outliers. Third, P10 minimizes MAE for several configurations but degrades $\delta_{1}$ (ViT-S P10 0.9421 vs. mean 0.9785). The arithmetic mean is therefore adopted for deployment (ViT-L Stage 4: MAE = 0.1059 m, RMSE = 0.1484 m, AbsRel = 0.0553, $\delta_{1}=0.9898$). Percentile estimators favor foreground depth and can reduce MAE at the cost of threshold accuracy; aggregation choice is therefore part of the evaluation protocol rather than a post hoc detail.

### 5.2. Training Curriculum Ablation

Table 16 evaluates the effect of progressive curriculum training on the NYU Depth V2 Eigen test split. The ablation isolates the contribution of each training stage by incrementally extending the supervision pipeline from synthetic metric pretraining to real indoor adaptation.

The untrained relative-depth ViT-S model performs poorly under metric evaluation, achieving only $\delta_{1}=0.385$ after median scale alignment. This confirms that relative monocular depth representations alone are insufficient for reliable metric distance estimation. In contrast, the Hypersim-pretrained metric model substantially improves performance to $\delta_{1}=0.817$ with AbsRel reduced from 0.437 to 0.143. The large gain demonstrates that synthetic metric supervision provides a strong geometric prior for indoor scene understanding and establishes an initial metric scale calibration before adaptation to real imagery data.

However, a substantial synthetic-to-real domain gap remains after Hypersim training alone. Although Hypersim provides dense, geometrically consistent metric supervision, its rendered appearance, noise characteristics, lighting distributions, and surface statistics differ from real RGB-D sensor data. Additional adaptation stages are therefore needed to handle sensor artifacts, missing depth regions, motion blur, clutter, and illumination variability common in real indoor environments. Training directly on NYU Depth V2 further improves accuracy. The NYU-only trained baseline initialized from relative depth reaches $\delta_{1}=0.854$, while initialization from the Hypersim metric checkpoint increases performance to $\delta_{1}=0.872$ and reduces RMSE from 0.603 m to 0.539 m. This indicates that synthetic metric pretraining remains beneficial even after supervised optimization on the target dataset.

**Figure 7:**
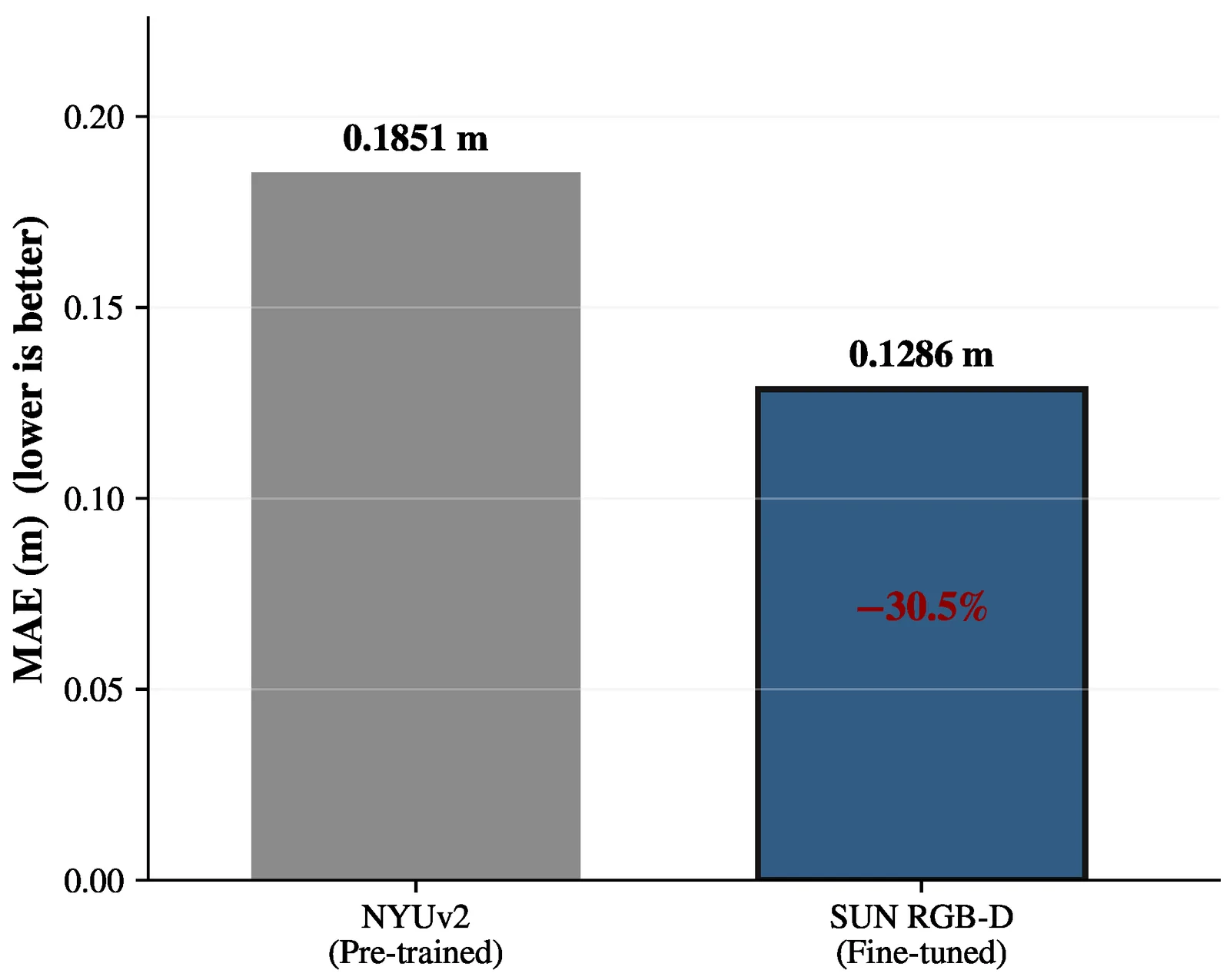
Effect of target-domain adaptation. Object-wise MAE on frozen SUN RGB-D test pairs (ViT-S, mean aggregation, n = 7876, conf = 0.45, IoU = 0.50). The NYUv2 pre-trained model achieves a baseline MAE of 0.1851 m. After fine-tuning on the target domain (SUN RGB-D), the MAE improves to 0.1286 m, corresponding to a 30.5% relative reduction. The dark blue bar with the black outline represents the deployed checkpoint utilized for all downstream evaluations.

The largest improvement is obtained when Hypersim pretraining is followed by NYU fine-tuning, yielding $\delta_{1}=0.916$ and reducing RMSE to 0.389 m. Introducing the intermediate ScanNet stage further improves performance to $\delta_{1}=0.929$ with AbsRel reduced to 0.0923. ScanNet acts as a domain-bridging stage between synthetic and real indoor imagery by exposing the model to real sensor noise and broader indoor scene variability before final calibration on NYU Depth V2. This progressive adaptation stabilizes the transition from synthetic supervision to real-world deployment conditions. The curriculum also improves transferability to SUN RGB-D, which contains more diverse scene layouts, cluttered environments, and heterogeneous capture conditions than NYU Depth V2. By gradually adapting the model from synthetic metric supervision to increasingly challenging real indoor datasets, the training pipeline improves both metric consistency and robustness for downstream per-object distance estimation. Finetuning on SUN RGB-D reduces MAE by 30.5% compared to prior NYUv2 stage (Figure 7).

The ViT-B backbone achieves the highest overall accuracy with $\delta_{1}=0.941$ and SiLog of 0.104, indicating that the curriculum remains effective across model scales. Overall, the results show that progressive multi-stage indoor training consistently improves metric depth estimation relative to single-stage or direct target-only optimization. The curriculum establishes large-scale geometric priors through synthetic supervision before progressively adapting the model to real indoor scene statistics, sensor characteristics, and target-domain metric calibration.

### 5.3. Surface Centroid Discrepancy

Surface-based and centroid-based object distances follow different geometric references (Section 2.2) and are not interchangeable as evaluation ground truth even when both are valid within their task domains.

**Figure 8:**
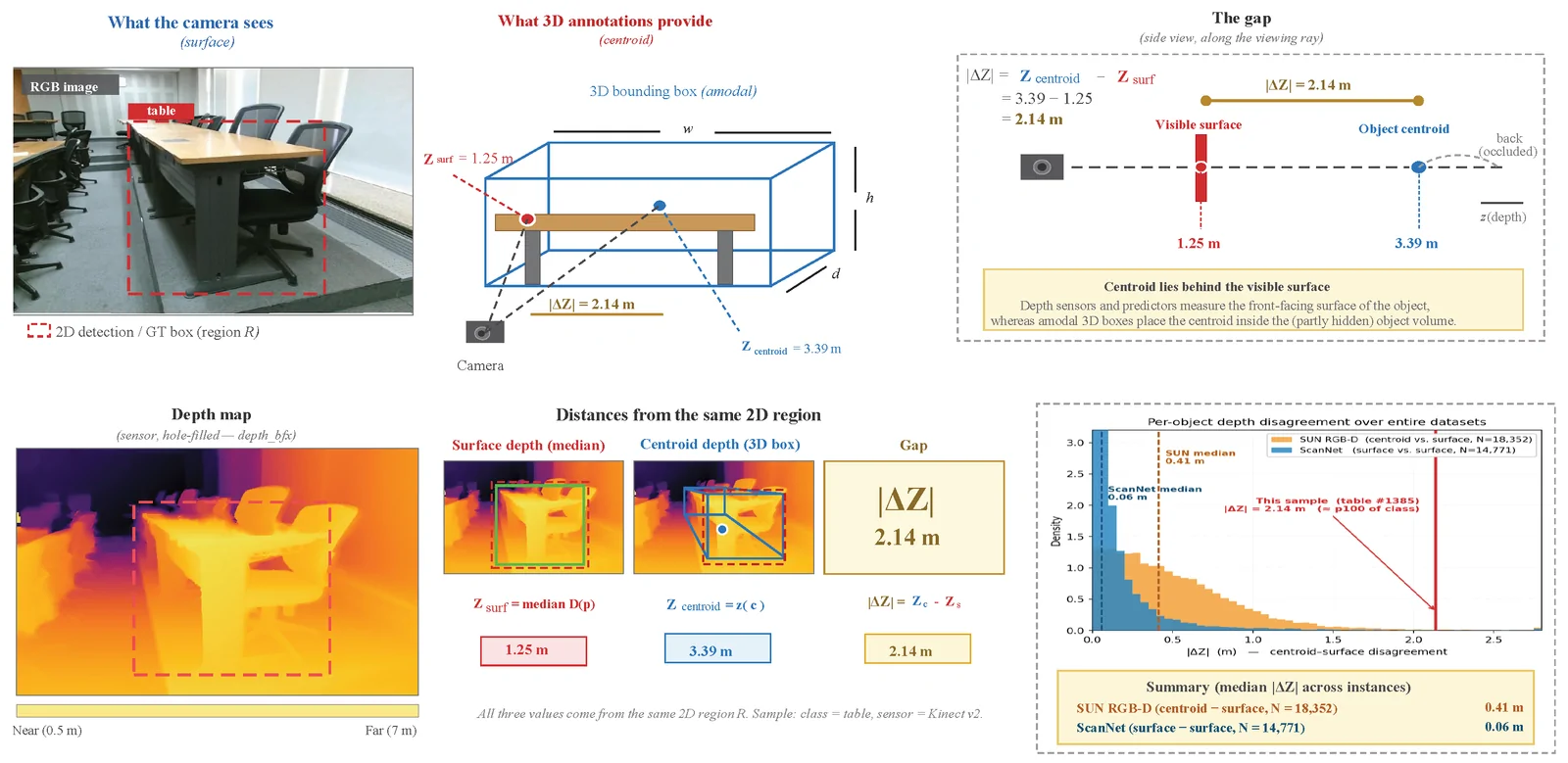
Geometric relationship between depth to the visible surface within a 2D region and depth to the object centroid implied by amodal 3D annotation, illustrated with a representative indoor instance and summarized with dataset-level distributions. The discrepancy |ΔZ| quantifies the bias induced when surface-aligned depth predictions are evaluated against centroid-based references.

To ground this discussion quantitatively, we measure the absolute offset $\Delta Z=|Z_{\mathrm{surf}}-Z_{\mathrm{centroid}}|$ across 18,352 annotated instances of the ten canonical indoor object classes in SUN RGB-D (illustrated in Figure 8), using the official hole-filled depth maps. $Z_{\mathrm{surf}}$ is computed as the median of valid GT depth pixels inside the annotated 2D bounding box, and $Z_{\mathrm{centroid}}$ is read from the 3D bounding box centroid annotation. Both quantities are computed independently of any model prediction. Table 17 reports per class statistics.

Across 18,352 SUN RGB-D instances, the median surface-to-centroid offset is 0.414 m (Table 17), exceeding typical same-box MAE and confirming that centroid-based references are not interchangeable with surface aggregation. Full per-class, per-sensor, and histogram analyses are provided in Appendix H.

## 6. Computational Efficiency

All runtime benchmarks are measured on an NVIDIA A40 GPU using the primary 518×518 input resolution and batch size 1. The reported deployment configuration uses the smallest backbone variants for both tasks (Depth Anything V2 ViT-S and YOLO11n), targeting real-time inference on consumer and server GPUs. The FPS and Latency results are measured on an NVIDIA A40 GPU. Extending the pipeline to edge devices with limited computational resources may require further optimization. The reported end-to-end throughput includes the complete late-fusion pipeline, i.e., monocular depth estimation, YOLO-based object detection, and per-box distance aggregation. Under the deployment configuration where only the depth branch is executed in FP16 mixed precision (while the detector and aggregation modules remain in FP32), the full pipeline achieves 19.42 ms latency per frame, corresponding to 51.5 FPS. Runtime evaluation follows a fixed benchmarking protocol consisting of 5 warm-up iterations followed by 50 timed iterations. Only model inference time is included in the reported latency; disk I/O, image loading, and preprocessing overheads are excluded.

Under FP32 execution, the depth branch requires 40.17 ms at 518×518 resolution on an NVIDIA A40 GPU, the detection branch adds a constant 7.83 ms, and the aggregation module contributes 0.80 ms, yielding a full pipeline latency of 52.25 ms (19.1 FPS). Converting only the depth branch to FP16 mixed precision reduces depth latency to 10.79 ms, a factor of 3.73 speedup with no accuracy loss on NYU Depth V2 ($\delta_{1}=0.9286$ under FP16 versus 0.9285 under FP32). Detection remains at 7.83 ms because its FP32 latency is already small and because retaining FP32 avoids any risk of detection accuracy regression. With selective FP16 acceleration, the full pipeline runs at 19.42 ms latency (51.5 FPS) on the A40 GPU. These benchmarks are specific to a server-grade NVIDIA A40 data-center GPU and should not be extrapolated to edge or embedded platforms without platform-specific profiling and re-optimization. Under selective FP16, the measured 19.42 ms latency (51.5 FPS) demonstrates real-time throughput on this server-class GPU; consumer GPUs with similar tensor-core support are expected to achieve interactive rates, whereas edge devices with limited compute may require model compression or hardware-specific tuning. Table 18 summarizes the breakdown at the primary resolution for the end-to-end pipeline, while Table 19 presents complete efficiency benchmarks for the depth branch across three backends and six resolutions on an NVIDIA A40 GPU (FP32, FP16) and CPU (dynamic INT8), showing the effect of resolution and backend choice on computational efficiency.

## 7. Discussion

The same-box protocol reads predicted and reference distances from the same predicted bounding box, matching what a deployed late-fusion system can compute. Under this protocol on SUN RGB-D, the curriculum-trained Depth Anything V2 ViT-S model reaches MAE = 0.1286 m and $\delta_{1}\;=\;0.9785$ on 7876 matched pairs (Table 9); ViT-L reduces MAE to 0.1059 m. Most of this gain comes from SUN RGB-D adaptation after the ScanNet and NYU stages, whereas zero-shot pretrained models show substantially higher error without target-domain fine-tuning (Table 6, Table 7 and Table 8). Under the indoor curriculum and fixed operating point, curriculum-trained Depth Anything V2 reports lower object-distance MAE than Depth Pro in the benchmark comparison; ViT-S yields the lowest latency among evaluated backbones at comparable object-distance accuracy under the reported protocol. Detection quality sets the spatial support for depth aggregation. Protocol validation (Table 14) shows that same-box and oracle-box references differ by only 0.0003 m MAE, whereas deliberately mismatched regions add 7.2%. Per-class analysis (Figure 5) links higher distance error to low recall on rare or ambiguous categories rather than to depth calibration alone. Mean box aggregation yields lower MAE than center-pixel sampling on hole-filled ground truth under the same protocol (Table 15). The surface-to-centroid offset (median 0.414 m, Table 17) exceeds typical per-object MAE, so comparing surface-based late fusion against centroid-based 3D references mixes annotation geometry with estimation error. On a server-grade NVIDIA A40 GPU, the selective FP16 pipeline runs at 51.5 FPS (19.42 ms per frame, Table 18), with preprocessing excluded; these throughput figures should not be extrapolated to edge or embedded platforms without platform-specific profiling. The same-box protocol applies to any RGB-D dataset with registered metric depth once class vocabularies are aligned for the matching step.

## 8. Limitations and Future Directions

The reported results are subject to several limitations. First, all distance evaluations are carried out on the official pre-processed SUN RGB-D ground-truth depth maps. The results demonstrate that statistical mean over region of interest outperforms median for metric distance aggregation. The behavior of these aggregation methods may change due to the depth distribution properties when tested on raw depth maps. Second, evaluation is conducted exclusively on SUN RGB-D, focusing on indoor scenes. Although the same-box protocol is dataset-agnostic, the operating-point selection (conf = 0.45, IoU = 0.50, 8 m depth cap) is calibrated to SUN RGB-D. Zero-shot depth evaluation on DIODE indoor is substantially weaker (AbsRel =0.307, $\delta_{1}=0.381$), so domain-specific adaptation is needed before deploying the depth branch on other datasets. Third, the object detector is restricted to ten canonical SUN RGB-D classes, which enables controlled evaluation but limits overall coverage. The full dataset contains over a thousand categories with strong long-tail imbalance; therefore, the canonical ten classes provide a trade-off between coverage and training feasibility. Fourth, supervised metric depth models inherit limitations of the underlying sensing modalities, including restricted range, missing data, quantization, and noise near reflective or translucent surfaces. Monocular depth therefore complements, but does not replace, calibrated sensing in precision-critical scenarios. Fifth, the same-box protocol and reported results target surface-based distance aggregation from dense depth maps rather than centroid distances from 3D bounding boxes.

## 9. Conclusions

In this work, we defined an accurate detections-conditioned, same-box evaluation protocol for dense depth-based per-object distance estimation in indoor scenes. We implemented it using a modular late-fusion framework that combines YOLO11n object detection with Depth Anything V2 monocular metric depth estimation. The experimental results show that the metric distance aggregated from a selected region of interest is highly dependent on the depth distribution within that region. For instance, mean, median and p10 (10th percentile) correspond to different final metric distances under identical conditions. Hence, spatial region selection, reference signal, predicted depth and the statistical distance aggregation method within the selected region of interest are essential components of the dense-depth-based distance prediction. Under the proposed protocol with mean aggregation and pre-processed ground truth, the ViT-L model achieved the best performance, reducing the mean absolute error to 0.1059 m while attaining a $\delta_{1}$ accuracy of 0.9898 on 7876 matched object instances. With selective FP16 depth inference, the full pipeline with ViT-S achieves 19.42 ms latency (51.5 FPS) on an NVIDIA A40 GPU. We further demonstrated that surface-based distances derived from bounding-box depth aggregation differ systematically from 3D centroid distances, with a median discrepancy of 0.414 m on SUN RGB-D (Table 17), highlighting the importance of clearly defining geometric reference points when evaluating object-level distance estimation methods.

In conclusion, the study establishes a practical and reproducible baseline for indoor object-level distance estimation and highlights the importance of clearly separating geometric reference definitions from aggregation-induced variability in evaluation.

## Figures and Tables

**Table 1:** Comparison of per-object distance estimation approaches across evaluation dimensions. The same-box protocol introduced in this paper is distinct from all prior evaluation designs. Bold shows evaluation settings for our method.

Method	Domain	Range	GT Reference	Aggregation Region
Zhu & Fang [12]	Outdoor	5–80 m	LiDAR centroid	End-to-end regressor
DistFormer [11]	Outdoor	5–80 m	GT mask median	GT segmentation mask
DECADE [13]	Outdoor	0–150 m	LiDAR range	YOLO + MLP regressor
Cube R-CNN [27]	Indoor	Indoor	3D box IoU	Volumetric AP3D
**Ours**	**Indoor**	**0.1–8 m**	**Same-box GT depth**	**Predicted box**

**Table 2:** Four-stage supervised depth training curriculum. At each stage, the model is exposed to progressively narrower but more task-relevant distributions, establishing a geometric prior before calibrating to the metric depth scale of the target domain.

Stage	Dataset	Role	Range (m)	Pairs
1	Hypersim	Synthetic geometric prior	0.1–20	59.5 K
2	ScanNet	Real indoor domain bridge	0.1–10	19.5 K
3	NYU Depth V2	Metric scale calibration	0.1–10	43.2 K
4	SUN RGB-D	Target domain adaptation	0.1–8	4.8 K

**Table 3:** YOLO11n fine-tuning on SUN RGB-D (Ultralytics [35]).

Setting	Value
Architecture/init	YOLO11n; COCO-pretrained
Input size	640×640
Epochs	150
Batch (per device)	16 (nominal batch 64)
Optimizer	AdamW; ${\mathrm{lr}}_{0}=0.01$; cosine lrf=0.01
Weight decay	$5\times 10^{-4}$
Early stopping patience	15 epochs
AMP/seed	enabled; seed 0

**Table 4:** Supervised monocular depth estimation on NYU Depth V2 (Eigen split, 0–10 m). Arrows indicate whether higher (↑) or lower (↓) is better.

Method	Backbone	Parameters Count	$\delta_{1}$↑	AbsRel ↓	RMSE ↓
BTS [41]	ResNet-50	47 M	0.885	0.110	0.392
AdaBins [18]	EfficientNet-B5	78 M	0.903	0.103	0.364
GLPDepth [42]	ResNet-50	58 M	0.900	0.102	0.360
NeWCRFs [20]	Swin-L	110 M	0.922	0.095	0.334
DPT [3]	ViT-hybrid	123 M	0.904	0.109	0.357
BinsFormer [43]	Swin-L	110 M	0.907	0.099	0.357
UniDepth v2 [4]	ViT-L	–	0.988	0.046	0.180
Metric3D v2 [23]	ViT-L	1.378 G	0.989	0.047	0.183
DA V2 ViT-L * [1]	DINOv2-L	335.3 M	**0.984**	0.056	0.206
DA V2 ViT-S * [1]	DINOv2-S	25 M	**0.961**	**0.073**	**0.261**
Ours-ViT-S (FP32)	DINOv2-S	25 M	0.9285	0.0923	0.3629
**Ours (FP16)** ★	DINOv2-S	25 M	**0.9286**	**0.0922**	**0.362**
Ours-ViT-B (FP32)	DINOv2-B	97.5 M	0.941	0.0934	0.360
Ours-ViT-L	DINOv2-L	335.3 M	0.9493	0.0871	0.3458

* Depth Anything V2 uses large-scale pretraining on mixed indoor and outdoor, real and synthetic data, whereas the proposed curriculum is restricted to indoor datasets and is optimized for downstream per-object distance evaluation rather than single-benchmark performance. ★ indicates the deployment-aligned efficient model.

**Table 5:** Metric depth evaluation on SUN RGB-D. Arrows indicate whether higher (↑) or lower (↓) is better.

Backbone	Training Condition	$\delta_{1}$↑	$\delta_{2}$↑	$\delta_{3}$↑	AbsRel ↓	SqRel ↓	RMSE (m) ↓	${\mathrm{RMSE}}_{\log}$↓
ViT-S	SUN RGB-D only baseline	0.839	0.944	0.968	0.318	1.031	1.110	0.194
ViT-S	Training Stage 4	0.881	0.963	0.980	0.149	0.176	0.370	0.190
ViT-L	Training Stage 3	0.859	0.960	0.977	0.202	0.370	0.431	0.218
ViT-L	Training Stage 4	0.916	0.970	0.982	0.136	0.186	0.334	0.172

**Table 6:** Zero-shot object distance estimation on the SUN RGB-D dataset with mean pooling inside each predicted box. All models evaluate on n=7876 matched pairs from a single fixed detection run. Best results per metric are bolded. Arrows indicate whether higher (↑) or lower (↓) is better.

Model	Encoder	MAE ↓	RMSE ↓	AbsRel ↓	SqRel ↓	$\delta_{1}$↑
UniDepth2 [4]	ViT-S	0.523	0.595	0.280	0.179	0.463
	ViT-B	0.562	0.622	0.298	0.192	0.373
	ViT-L	0.570	0.622	0.301	0.189	0.330
Metric3D v2 [23]	ViT-S	**0.411**	**0.479**	**0.220**	**0.115**	**0.656**
	ViT-L	0.452	0.504	0.239	0.125	0.585
DA2 Hypersim [1]	ViT-S	0.858	0.968	0.453	0.460	0.181
	ViT-B	0.893	0.992	0.468	0.478	0.137
	ViT-L	0.897	0.985	0.470	0.471	0.108
Depth Pro [2]	—	0.584	0.694	0.310	0.241	0.433

**Table 7:** Zero-shot object distance estimation on the SUN RGB-D dataset with median pooling inside each predicted box. All models evaluate on n=7876. Best results per metric are bolded. Arrows indicate whether higher (↑) or lower (↓) is better.

Model	Encoder	MAE ↓	RMSE ↓	AbsRel ↓	SqRel ↓	$\delta_{1}$↑
UniDepth2 [4]	ViT-S	0.511	0.587	0.285	0.180	0.464
	ViT-B	0.548	0.610	0.301	0.190	0.371
	ViT-L	0.546	0.600	0.299	0.182	0.344
Metric3D v2 [23]	ViT-S	**0.404**	**0.473**	**0.225**	**0.116**	**0.639**
	ViT-L	0.437	0.486	0.241	0.121	0.580
DA2 Hypersim [1]	ViT-S	0.818	0.932	0.449	0.440	0.196
	ViT-B	0.848	0.952	0.460	0.452	0.156
	ViT-L	0.850	0.943	0.461	0.443	0.130
DepthPro [2]	—	0.561	0.676	0.309	0.234	0.440

**Table 8:** Zero-shot object distance estimation on the SUN RGB-D dataset with 10th percentile (p10) pooling inside each predicted box. All models evaluate on n=7876. Best results per metric are bolded. Arrows indicate whether higher (↑) or lower (↓) is better.

Model	Encoder	MAE ↓	RMSE ↓	AbsRel ↓	SqRel ↓	$\delta_{1}$↑
UniDepth2 [4]	ViT-S	0.454	0.539	0.333	0.205	0.393
	ViT-B	0.472	0.547	0.340	0.204	0.324
	ViT-L	0.467	0.535	0.334	0.193	0.300
Metric3D v2 [23]	ViT-S	**0.365**	**0.438**	**0.270**	**0.139**	**0.535**
	ViT-L	0.387	0.443	0.282	0.139	0.456
DA2 Hypersim [1]	ViT-S	0.691	0.801	0.497	0.428	0.191
	ViT-B	0.695	0.800	0.493	0.417	0.168
	ViT-L	0.692	0.790	0.488	0.403	0.148
Depth Pro [2]	—	0.474	0.583	0.339	0.227	0.404

**Table 9:** Primary per-object distance estimation results under the fixed same-box protocol (Training stage 4 (Hypersim + ScanNet + Nyu + SUN RGB-D)—Final results). Bold shows best aggregation method for each of the MDE model variant. Arrows indicate whether higher (↑) or lower (↓) is better.

Backbone	Aggregation	MAE (m) ↓	RMSE (m) ↓	AbsRel ↓	$\delta_{1}$↑	Pairs
Depth Anything V2 ViT-S	Mean	**0.1286**	**0.1817**	**0.0662**	0.9785	7876
	Median	0.1319	0.1895	0.0713	**0.9716**	7876
	P10	0.1282	0.1962	0.0960	0.9421	7876
Depth Anything V2 ViT-B	Mean	**0.1087**	**0.1547**	**0.0557**	0.9886	7876
	Median	0.1101	0.1615	0.0591	**0.9836**	7876
	P10	**0.1064**	0.1727	0.0786	0.9611	7876
Depth Anything V2 ViT-L	Mean	**0.1059**	**0.1484**	**0.0553**	0.9898	7876
	Median	0.1096	0.1576	0.0598	**0.9851**	7876
	P10	0.1065	0.1725	0.0804	0.9641	7876
Depth Pro (native)	Mean	**0.1615**	**0.2084**	**0.0879**	**0.9718**	7876
	Median	0.1688	0.2192	0.0960	0.9548	7876
	P10	0.1717	0.2326	0.1322	0.9024	7876

Pairs: fixed matched true-positive detections (n=7876; conf =0.45, IoU =0.50), identical across backbones and aggregations.

**Table 10:** Per-object distance estimation results (training stage 3—Hypersim + ScanNet + Nyu), under the same conditions as Table 9. Arrows indicate whether higher (↑) or lower (↓) is better.

Backbone	Aggregation	MAE (m) ↓	RMSE (m) ↓	AbsRel ↓	$\delta_{1}$↑	Pairs
Depth Anything V2 ViT-S	Mean	0.1851	0.2511	0.0942	**0.9344**	7876
	Median	0.1873	0.2555	0.0996	0.9229	7876
	P10	**0.1733**	**0.2417**	**0.1260**	0.8787	7876
Depth Anything V2 ViT-B	Mean	0.1676	0.2217	**0.0859**	**0.9580**	7876
	Median	0.1674	0.2224	0.0901	0.9487	7876
	P10	**0.1608**	**0.2228**	0.1173	0.9091	7876
Depth Anything V2 ViT-L	Mean	0.1732	0.2307	**0.0888**	**0.9507**	7876
	Median	0.1734	0.2327	0.0932	0.9398	7876
	P10	**0.1613**	**0.2263**	0.1180	0.8977	7876
Depth Pro (native)	Mean	0.4861	0.5397	0.2642	0.4580	7876
	Median	0.4842	0.5408	0.2736	0.4383	7876
	P10	**0.4491**	**0.5107**	**0.3361**	0.3343	7876

**Table 11:** GT vocabulary composition and image-level statistics on the SUN RGB-D test split (5050 images; conf = 0.45, IoU = 0.50). Object-level percentages are relative to all 29,567 GT objects; image-level percentages are relative to 5050 test images.

Quantity	Value (% of Total)
Object-Level	
Total GT objects	29,567 (100%)
Mapped to 10 canonical training classes	18,352 (62.1%)
Out-of-vocabulary (OOV) objects	11,215 (37.9%)
Image-Level (*n* = 5050)	
Images with any canonical GT object	4255 (84.3%)
Images with only OOV GT objects	773 (15.3%)
Images with no GT annotation	22 (0.4%)

**Table 12:** Detector operating point Object counts at (conf = 0.45, IoU = 0.50). The canonical ground truth total is 18,352 objects. Percentages are relative to this canonical total.

Detection Outcome	Count (% of Canonical GT)
False Negatives (FN)	10,476 (57.1%)
Matched True Positives (TP)	7876 (42.9%)
False Positives (FP)	2052

Total: TP + FN = 7876 + 10,476 = 18,352 canonical GT objects.

**Table 13:** Object detection metrics for YOLO11n trained on SUN RGB-D.

Context	Metric	Value	Split
Training eval	mAP@50	0.878	val
(mAP, swept)	mAP@50–95	0.743	val
Deployment	Precision	0.793	test
operating point	Recall	0.429	test
(conf = 0.45)	F1	0.557	test

**Table 14:** Protocol validation under three evaluation conditions on 7876 matched pairs. ${\hat{B}}_{i}$ is the predicted box and $B_{i}^{*}$ is the annotation box. Same-box $\left({\hat{B}}_{i},{\hat{B}}_{i}\right)$ and oracle-box $\left(B_{i}^{*},B_{i}^{*}\right)$ differ by only 0.0003 m in MAE when both maps share a common spatial support. The localization-stress condition $\left({\hat{B}}_{i},B_{i}^{*}\right)$ intentionally mismatches the prediction and reference regions, producing the expected MAE increase. Arrows indicate whether higher (↑) or lower (↓) is better.

Condition	Pred. Region	GT Region	MAE ↓	AbsRel ↓	$\delta_{1}$ ↑
Same-box (★)	${\hat{B}}_{i}$	${\hat{B}}_{i}$	0.1286	0.0662	0.9785
Oracle-box	$B_{i}^{*}$	$B_{i}^{*}$	0.1289	0.0663	0.9789
Localization stress	${\hat{B}}_{i}$	$B_{i}^{*}$	0.1379	0.0714	0.9714

**Table 15:** Box-wise depth aggregation ablation on SUN RGB-D under the primary operating point and eval setup (5050 images, 7876 matched pairs, confidence =0.45, IoU =0.50, same-box GT depth, official hole-filled ground truth depth maps, canonical ten GT classes, depth cap 8 m). Best values per column are bold. Arrows indicate whether higher (↑) or lower (↓) is better. We designate the Arithmetic Mean (★) as our deployed strategy. While the 10th percentile (P10) yields a marginally lower MAE, the Mean exhibits superior robustness across all other metrics (lowest RMSE, AbsRel, SqRel, and highest $\delta_{1}$), making it the most statistically reliable choice.

Metric (↓/↑)	Median	Center	P10	Mean (★)
MAE (m) ↓	0.1319	0.1618	**0.1282**	0.1286
RMSE (m) ↓	0.1895	0.2398	0.1962	**0.1817**
AbsRel ↓	0.0713	0.0930	0.0960	**0.0662**
SqRel ↓	0.0180	0.0308	0.0303	**0.0155**
$\delta_{1}$↑	0.9716	0.9345	0.9421	**0.9785**
$\delta_{2}$↑	0.9978	0.9910	0.9877	**0.9991**
$\delta_{3}$↑	0.9997	0.9967	0.9948	**0.9999**

**Table 16:** Training curriculum ablation on the NYU Depth V2 Eigen test set (n=654, depth [0.1,10.0] m, input resolution 518×518). Models selected for metric distance are in **bold**. Arrows indicate whether higher (↑) or lower (↓) is better.

Training	Setting	$\delta_{1}$↑	$\delta_{2}$↑	$\delta_{3}$↑	AbsRel ↓	RMSE ↓	SiLog↓
w/o training	Relative-ViT-S *	0.385	0.640	0.805	0.437	1.364	0.496
	Hypersim-ViT-S	0.817	0.963	0.991	0.143	0.514	0.150
Single dataset	NYU only (ViT-S) *	0.854	0.960	0.976	0.130	0.603	0.297
	NYU only (ViT-S)	0.872	0.963	0.978	0.121	0.539	0.242
Curriculum	Hypersim → NYU (ViT-S)	0.916	0.985	0.997	0.100	0.389	0.116
	Hypersim → ScanNet → NYU (ViT-S)	**0.929**	**0.989**	**0.997**	**0.092**	**0.363**	**0.110**
	Hypersim → ScanNet → NYU (ViT-B)	0.941	0.990	0.997	0.0934	0.360	0.104

* Relative checkpoint (median scale alignment). All other checkpoints are Hypersim fine-tuned versions.

**Table 17:** Surface to centroid depth gap $\Delta Z=|Z_{\mathrm{surf}}-Z_{\mathrm{centroid}}|$ on SUN RGB-D (18,352 instances, 10 canonical classes, GT depth). $Z_{\mathrm{surf}}$ is the median GT depth inside the annotated 2D box and $Z_{\mathrm{centroid}}$ is the annotated 3D box centroid depth. Deeper% is the fraction of instances where the centroid lies behind the visible surface. ${\bar{Z}}_{c}$ is the mean centroid depth.

Class	N	${\bar{Z}}_{c}$ (m)	$\Delta Z_{\mathrm{med}}$ (m)	$\Delta Z_{p95}$ (m)	Deeper%
toilet	150	1.33	0.149	0.52	41
bathtub	49	1.77	0.328	0.71	65
chair	10,896	2.38	0.358	1.15	83
table	2535	2.12	0.414	1.26	89
desk	1895	2.17	0.463	1.29	87
sofa	631	2.54	0.582	1.34	92
bookshelf	746	2.78	0.613	1.38	95
dresser	636	2.64	0.658	1.34	96
night_stand	281	3.26	0.694	1.24	98
bed	533	2.39	0.695	1.31	88
All	18,352	2.34	0.414	1.24	85

**Table 18:** Full pipeline latency breakdown at 518×518 on an NVIDIA A40 GPU. Only the depth branch is accelerated with FP16. Detection latency is constant across conditions.

Precision	Depth (ms)	Det. (ms)	Aggr. (ms)	Total (ms)	FPS
FP32 (all)	40.17	7.83	0.80	52.25	19.1
**FP16 (depth only)**	**10.79**	7.83	0.80	**19.42**	**51.5**

**Table 19:** Efficiency benchmarks of our depth branch on an A40 GPU (FP32/FP16) and CPU (PyTorch dynamic INT8). Latency in milliseconds (↓ indicates lower is better), FPS in frames per second (↑ indicates higher is better). Green denotes the primary operating point (518×518, FP16). **Bold** denotes best per resolution across all configurations.

Resolution	FP32 (CUDA)		FP16 (CUDA)		INT8 (CPU)	
	Lat (ms) ↓	FPS ↑	Lat (ms) ↓	FPS ↑	Lat (ms) ↓	FPS ↑
224×224	10.03	99.69	**7.21**	**138.67**	43.36	23.06
252×252	11.79	84.84	**7.36**	**135.93**	51.14	19.55
322×322	19.68	50.81	**7.67**	**130.34**	74.63	13.40
392×392	24.42	40.96	**9.78**	**102.26**	98.33	10.17
504×504	36.21	27.62	**14.32**	**69.85**	221.37	4.52
518×518	40.17	24.90	**10.79**	**92.69**	226.33	4.42

**Figure A1:**
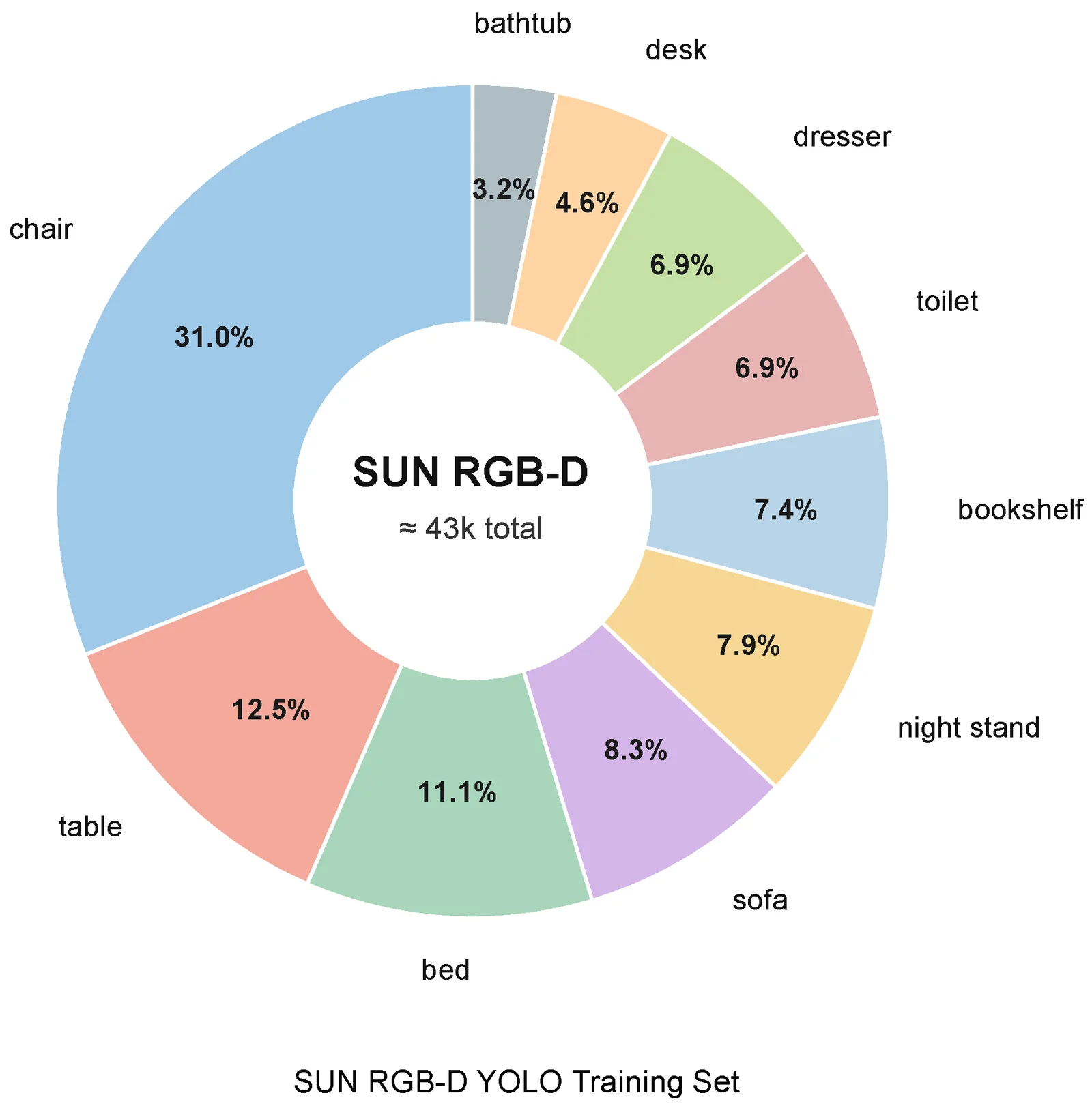
Ground truth instance count per class in the SUNRGB-D training set for object detection (YOLO11n training) before addressing class imbalance.

**Figure A3:**
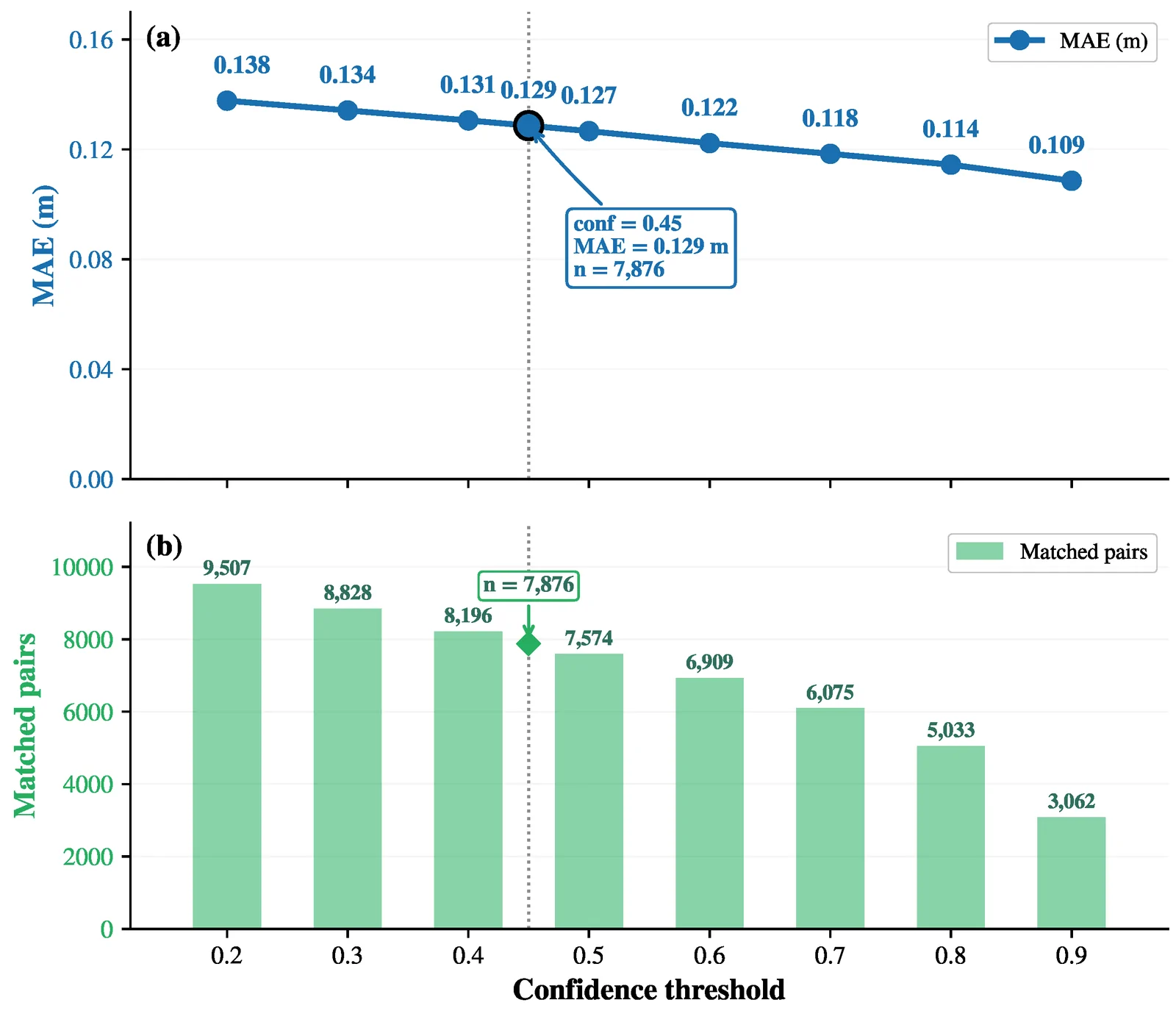
Confidence threshold and detection–estimation trade-off. (**a**) The MAE shows a monotonic decrease as the confidence threshold increases, proving that stricter filters yield more accurate depth estimates. (**b**) However, stricter confidence comes at the cost of discarding valid detected objects. The number of available matched pairs drops significantly from 9507 at conf = 0.2 to 3062 at conf = 0.9. The annotated operating point is conf = 0.45 (highlighted by the dotted line, black-outlined marker in a, and diamond marker in **b**). At this point, the system achieves a MAE = 0.129 m (0.1286 m) while retaining a sufficiently large and accurate matched pairs evaluation set of n = 7876 (IoU ≥ 0.50). We select this threshold to balance low depth error with adequate number of object’s coverage for downstream evaluation.

**Figure A7:**
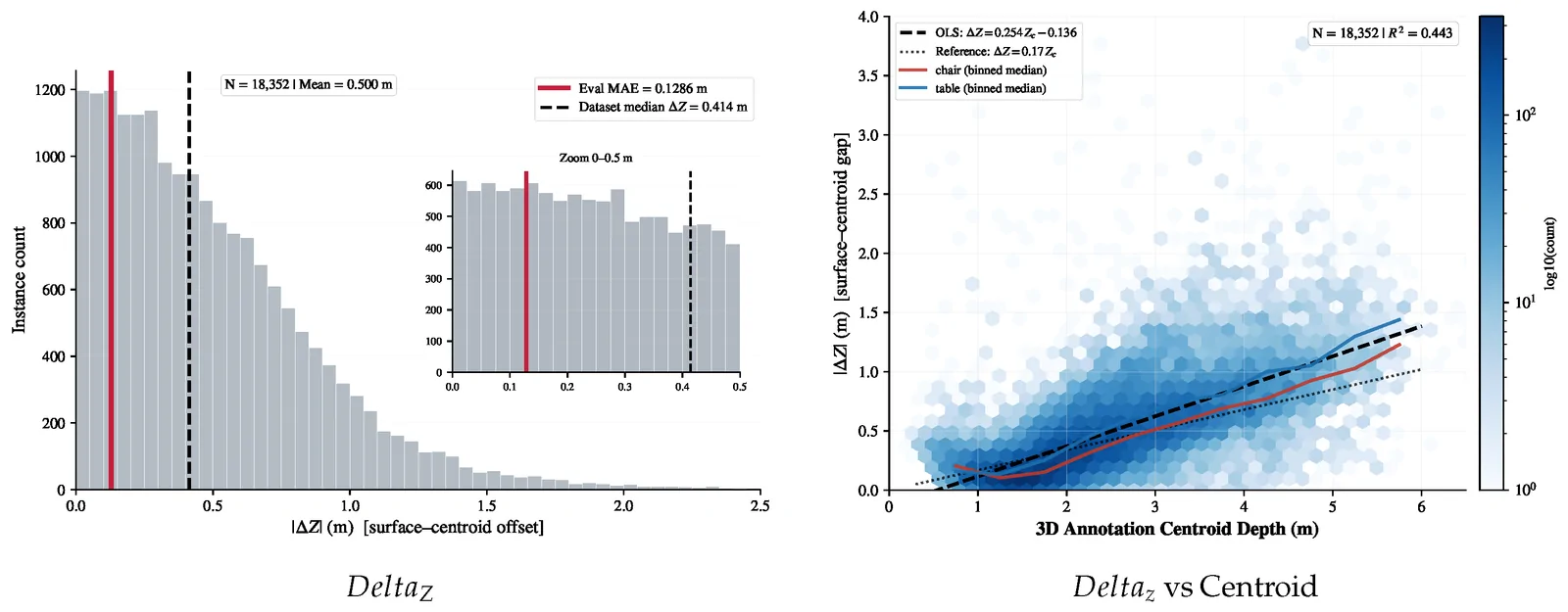
Surface-to-centroid depth gap in the SUN RGB-D test set. Histogram on left shows distribution of the absolute surface-to-centroid offset ΔZ across the 18,352 canonical-class annotated instances on the SUN RGB-D test split used in the frozen detector output evaluation. The vertical reference line marks the final system MAE under the same-box protocol using official hole-filled ground-truth depth (Depth Anything V2 ViT-S, mean aggregation: 0.1286 m). For more than 77% of instances, ΔZ alone exceeds this threshold, demonstrating that the surface-to-centroid gap is substantial relative to the achievable depth estimation accuracy. The hexbin density plot on the right illustrates the depth dependency of |ΔZ|. The offset exhibits a linear relationship as the object depth increases (OLS slope =0.254, $R^{2}=0.443$), indicating that estimated surface-centroid gaps grow systematically with distance. Binned medians for the dominant classes (chair and table) follow this trend closely.

**Figure A8:**
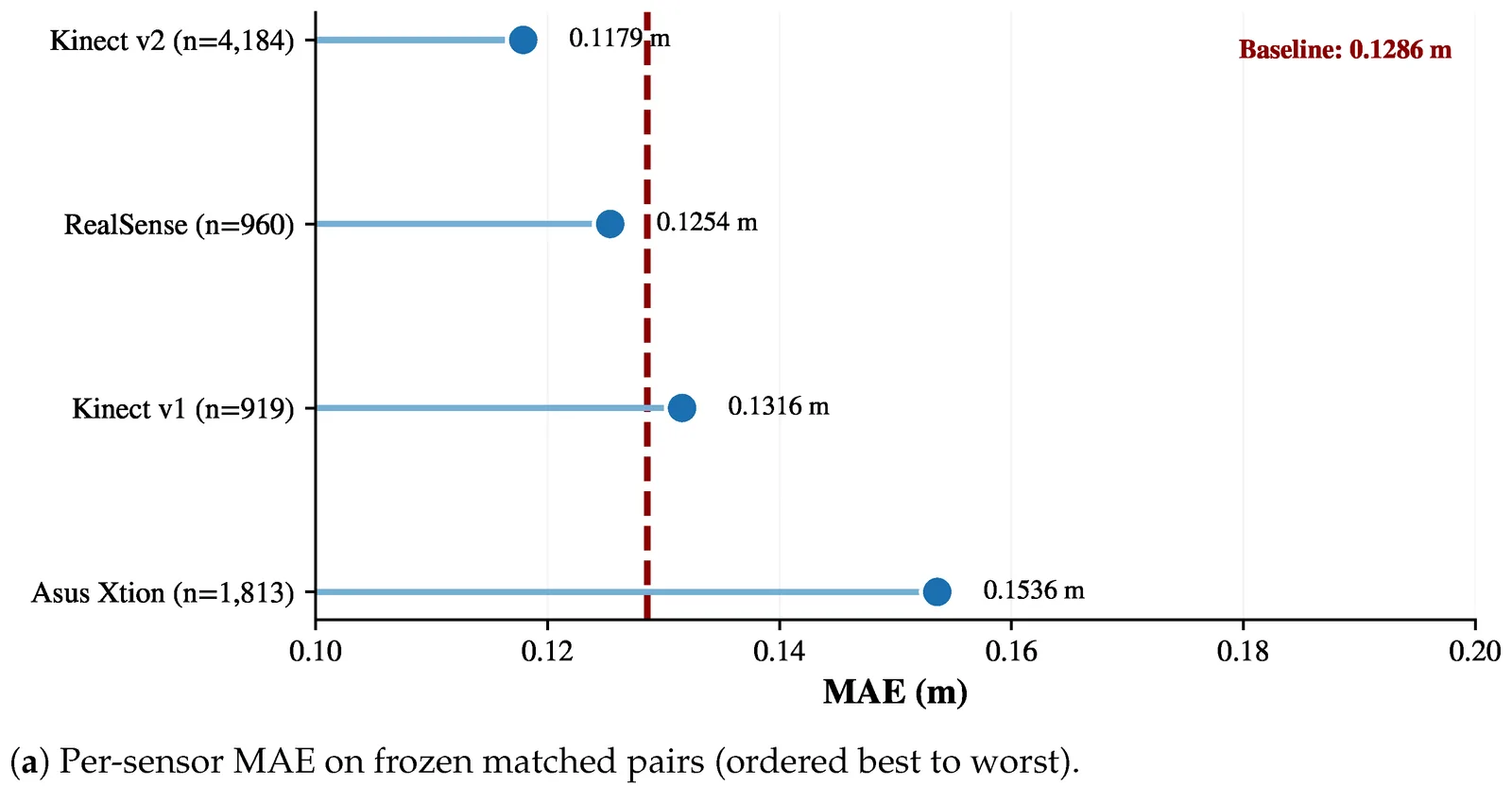

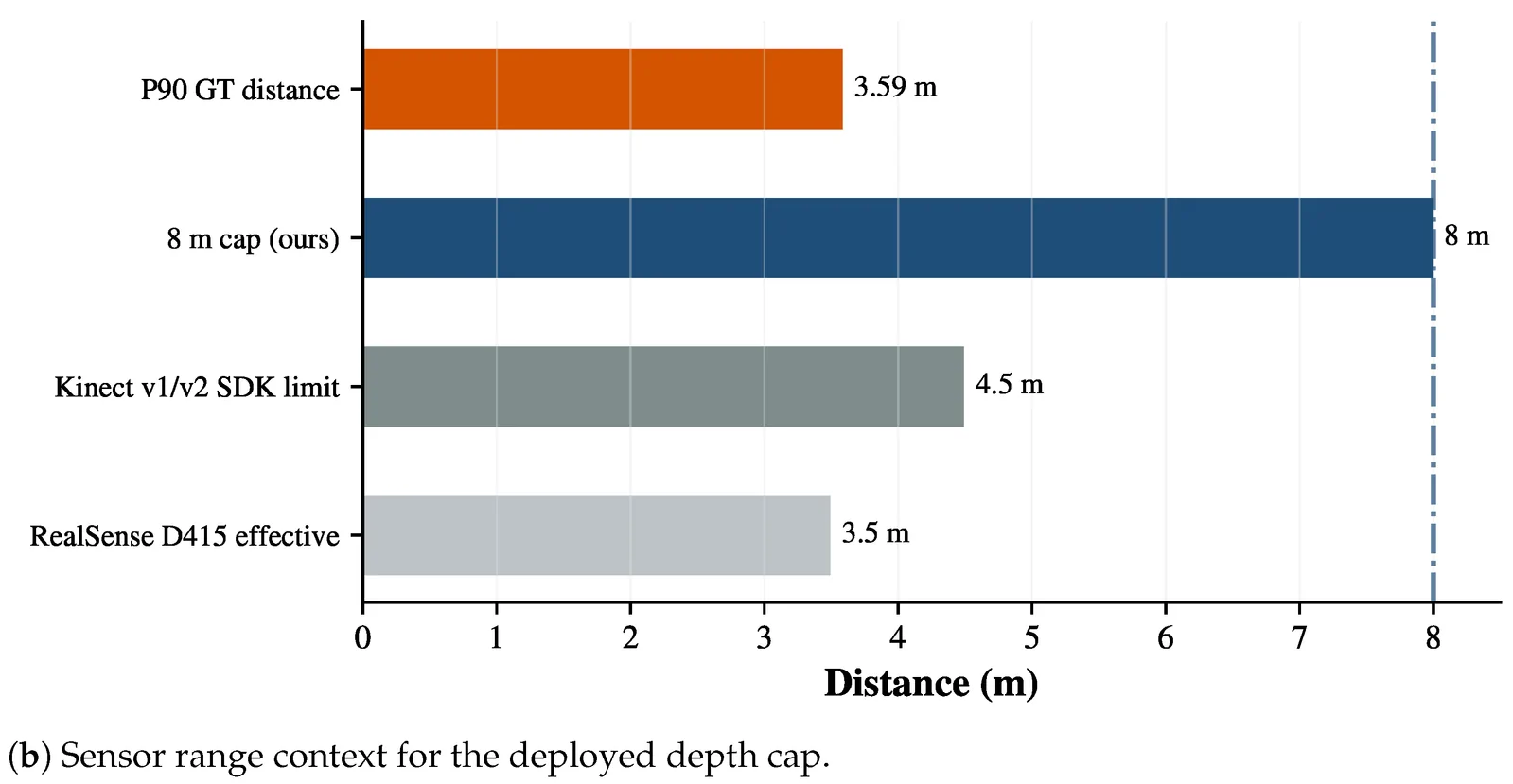
Hardware robustness and range context for depth estimation. (**a**) Our depth estimation pipeline is agnostic to the specific depth sensor used in the SUN RGB-D dataset, maintaining a consistently low MAE (<0.16 m) across Kinect v1/v2, RealSense, and Asus Xtion modalities. The baseline MAE of 0.1286 m (red dashed line) is derived from all n=7876 matched pairs. Kinect v2 yields the best results. (**b**) The deployed 8 m depth cap is mathematically justified i.e., the P90 object distance is only 3.59 m, comfortably inside the effective operating ranges of the actual sensors (3.5–4.5 m).

**Figure A9:**
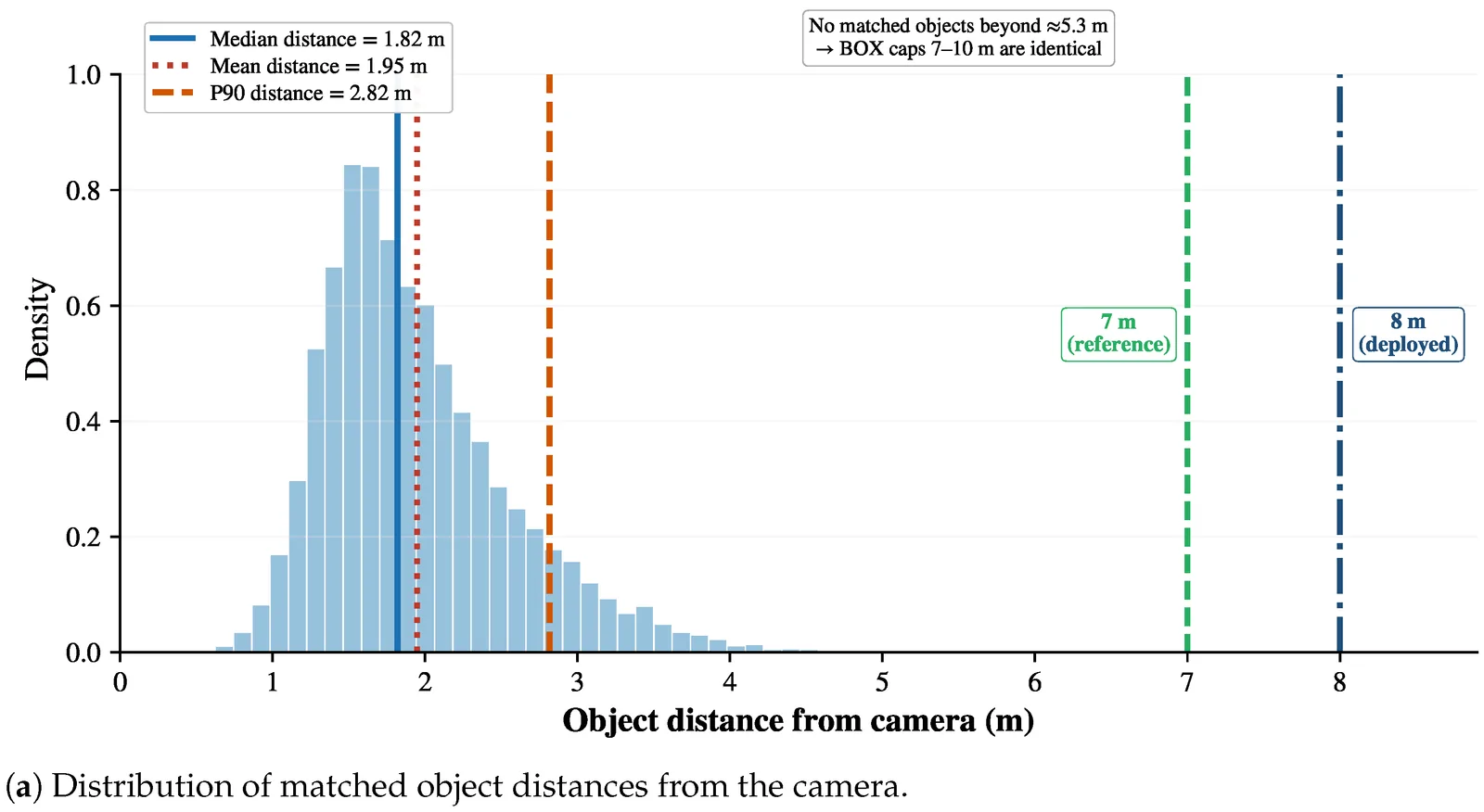

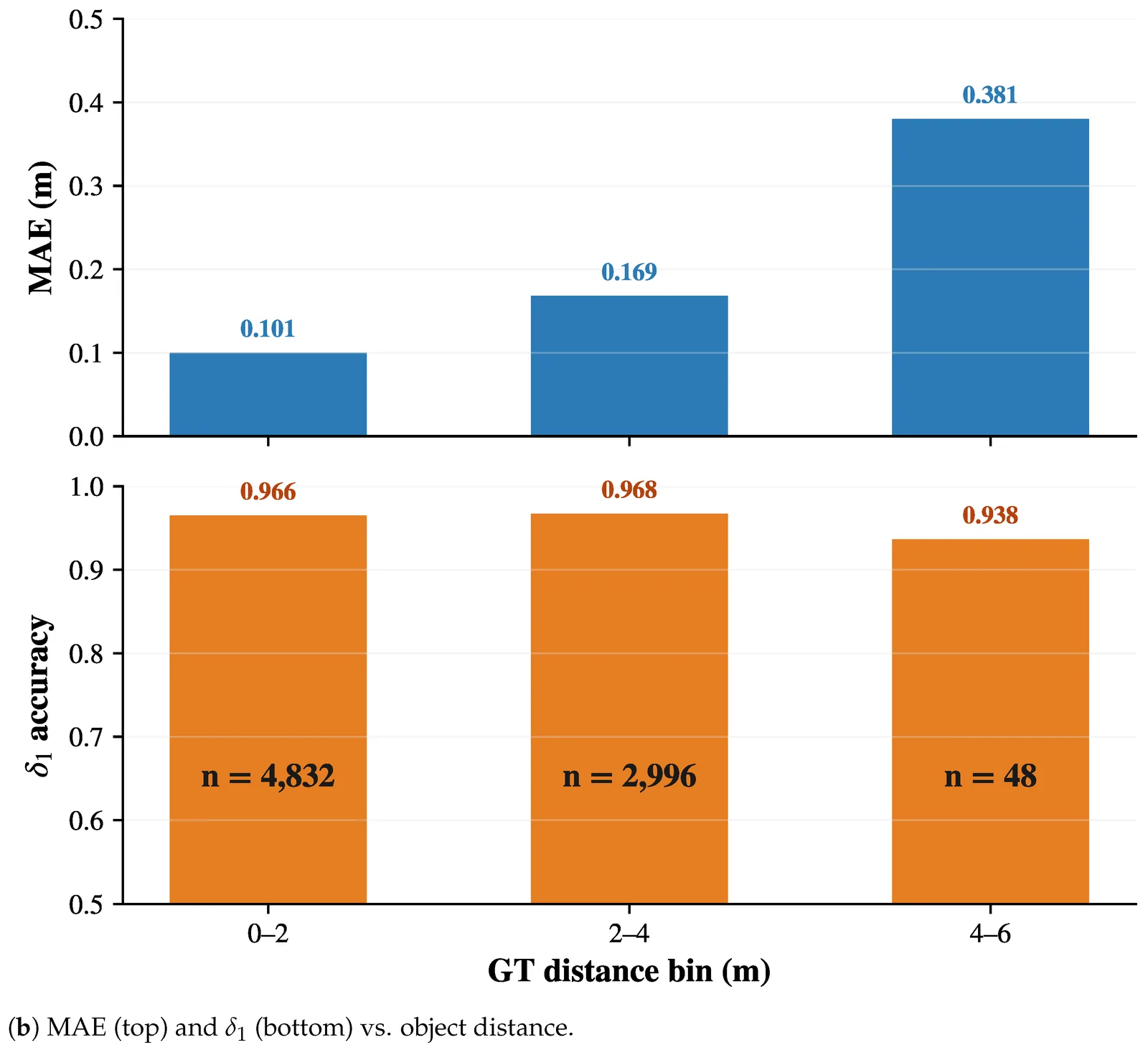
Distance range analysis of the SUN RGB-D test set. (**a**) Distribution of matched object distances extracted from the same-box ground-truth depth maps (surface depth, not 3D annotation centroids). Across the n=7876 matched pairs, the median and 90th percentile (P90) distances are 1.82m and 2.82m, respectively, with a maximum measured distance of ≈5.30m. (**b**) Depth estimation metrics stratified by ground-truth distance bins. The MAE and $\delta_{1}$ remain highly accurate for distances up to 4 m. Because the distribution in (**a**) tails off completely beyond 5.3m, the depth cap sweeps evaluated at 7m, 8m, and 10m all yield identical MAE values. This validates our deployment choice of an 8m cap as a conservative, precautionary clamp that safely exceeds the actual data range.

## Data Availability

The datasets used in this study are publicly available in the following repositories. The NYUv2 dataset is available at https://cs.nyu.edu/fergus/datasets/nyu_depth_v2.html (accessed on 23 July 2026). Hypersim is available at https://github.com/apple/ml-hypersim (accessed on 23 July 2026). ScanNet is available at http://www.scan-net.org/ (accessed on 23 July 2026) and its code repository at https://github.com/ScanNet/ScanNet (accessed on 23 July 2026). The SUNRGBD dataset is available at https://rgbd.cs.princeton.edu/ (accessed on 23 July 2026). All datasets were accessed and used in accordance with their respective licenses.

## Appendix A. Depth-Anything-V2 Performance on NYU Depth Without Additional Training

Table A1 reports Depth-Anything-V2 checkpoints evaluated without additional training in this project. The relative-depth checkpoints are evaluated with median scaling because they do not predict metric scale directly. The metric-Hypersim checkpoints are evaluated without median scaling.

Table A1 compares zero-shot indoor performance of the original Depth Anything V2 relative-depth checkpoints against the metric Hypersim-pretrained variants on the NYU Depth V2 benchmark. A clear performance gap is observed between relative and metric models across all backbone scales. The relative-depth checkpoints, despite using median scaling during evaluation, achieve limited indoor metric consistency, with the ViT-S model reaching only $\delta_{1}=0.3851$ and AbsRel = 0.4371. Even the larger ViT-L relative model improves only to $\delta_{1}=0.5192$, indicating that relative monocular depth representations alone do not generalize to calibrated indoor metric estimation reliably. In contrast, the metric Hypersim-pretrained checkpoints substantially improve all evaluation metrics without any additional indoor fine-tuning. The ViT-S metric model increases $\delta_{1}$ from 0.3851 to 0.8175 while reducing AbsRel from 0.4371 to 0.1432 and RMSE from 1.3640 m to 0.5146 m, demonstrating the strong effect of synthetic metric supervision. Similar trends are observed for ViT-B and ViT-L, where the metric variants consistently outperform their relative counterparts by large margins. The ViT-L metric checkpoint achieves the best overall zero-shot indoor performance with $\delta_{1}=0.8815$, AbsRel = 0.1262, and RMSE = 0.4741 m. The results indicate that metric pretraining on large-scale synthetic indoor data provides substantially better geometric calibration than relative-depth pretraining, and that increasing backbone capacity further improves zero-shot transfer to indoor metric benchmarks. Overall, the table validates the choice of the Hypersim metric checkpoint as the initialization point for the subsequent indoor curriculum training stages.

**Table A1:** Depth-Anything-V2 checkpoints evaluated on NYU Depth V2 without additional training on indoor data. Arrows indicate whether higher (↑) or lower (↓) is better.

Checkpoint	Backbone	Median Scale	$\delta_{1}$↑	AbsRel ↓	RMSE ↓	N
DA-V2 relative	ViT-S	Yes	0.3851	0.4371	1.3640	654
DA-V2 relative	ViT-B	Yes	0.4461	0.3748	1.2410	654
DA-V2 relative	ViT-L	Yes	0.5192	0.3241	1.0148	654
DA-V2 metric Hypersim	ViT-S	No	0.8175	0.1432	0.5146	654
DA-V2 metric Hypersim	ViT-B	No	0.8585	0.1317	0.4760	654
DA-V2 metric Hypersim	ViT-L	No	0.8815	0.1262	0.4741	654

Table 16 summarizes depth-only performance across the principal training stages. The relative-depth checkpoints are evaluated with median scaling because they do not predict metric scale directly. The metric-Hypersim checkpoints are evaluated without median scaling. The zero-shot relative baseline at 518 px achieves $\delta_{1}=0.385$, AbsRel = 0.437, and RMSE = 1.364 m. This performance is inadequate for reliable downstream object-distance estimation, confirming that domain-specific metric adaptation is necessary. The Hypersim-fine-tuned model achieves $\delta_{1}=0.81$ at the same resolution without further training. Fine-tuning directly on NYU from the Hypersim-metric checkpoint also yields $\delta_{1}=0.81$, whereas the staged mixed curriculum beginning with Hypersim synthetic pretraining followed by the indoor adaptation sequence produces the strongest results: $\delta_{1}=0.929$, AbsRel = 0.0922, RMSE = 0.3627 m. We adopt this checkpoint as the primary depth branch for all subsequent integrated evaluations because of its lower parameter count and relatively higher accuracy. The full curriculum outperforms direct single-stage NYU fine-tuning by 0.078 in $\delta_{1}$, validating the multi-stage training design.

## Appendix D. Detection Operating-Parameter Sensitivity

Figure A4 relates detection operating parameters to per-object distance MAE under hole-filled evaluation. At fixed IoU = 0.50 (left panel), MAE decreases from 0.136 m at conf = 0.25 to 0.124 m at conf = 0.55, but matched-pair count drops sharply beyond conf = 0.45, which we retain as the primary operating point. At fixed conf = 0.45 (right panel), MAE falls from 0.169 m (IoU 0.50–0.60) to 0.109 m (IoU 0.90–1.00); bin counts should sum to the frozen matched-pair total n=7876 after figure regeneration (current plot annotations may show a one-count off-by-one in the highest-IoU bin).

## Appendix E. Per-Class Detection Detail

Per-class detection precision, recall, and F1 on a stratified SUN RGB-D test subset (Figure A5).

## Appendix F. Per-Class Mean vs. Median Aggregation

Per-class distance MAE under mean versus median box-wise aggregation (Figure A6a).

## Appendix G. Zero-Shot Depth Generalization

To assess the generalization of the trained depth branch beyond the training distribution, the final curriculum checkpoint is evaluated on two held-out benchmarks not used at any stage of training: the DIODE indoor validation set and the DA-2K pairwise depth ranking dataset. Neither benchmark was accessible during curriculum design, operating point selection, or any hyperparameter decision, ensuring a clean zero-shot assessment. Table A2 reports the zero-shot depth generalization results. On DIODE indoor (325 images, GT clipped to [0.1,10.0] m), the model achieves AbsRel =0.307, SqRel =0.664, RMSE =1.420 m, and $\delta_{1}=0.381$. On DA-2K indoor (420 pairs, 195 images), it achieves a pairwise ordering accuracy of 78.33%.

**Table A2:** Zero-shot depth generalization on held-out indoor benchmarks. Neither benchmark was used at any stage of curriculum training. Arrows indicate whether higher (↑) or lower (↓) is better.

Benchmark	N	Metric	Ours
DIODE indoor [40]	325	AbsRel ↓	0.307
DIODE indoor [40]	325	$\delta_{1}$↑	0.381
DIODE indoor [40]	325	$\delta_{2}$↑	0.697
DIODE indoor [40]	325	$\delta_{3}$↑	0.829
DA-2K indoor [1]	420 pairs	Pairwise acc. ↑	78.33%

The most informative observation is the relationship between the threshold accuracy metrics. The $\delta_{1}$ threshold requires predictions to fall within a factor of 1.25 of ground truth, while $\delta_{2}$ and $\delta_{3}$ relax this to factors of 1.56 and 1.95, respectively. The large step from $\delta_{1}=0.381$ to $\delta_{2}=0.697$ indicates that the model’s depth predictions are geometrically structured and ordinally consistent, but carry a systematic offset from the correct absolute metric scale. This pattern is characteristic of a scale calibration failure rather than a failure of geometric understanding. The DA-2K pairwise ordering accuracy of 78.33% corroborates this interpretation; the model correctly identifies the closer object in nearly four out of five annotated pairs on scenes it has never seen, confirming that relative depth ordering generalizes well across sensor domains. The two zero-shot results therefore measure different properties of the model and should not be interpreted as a unified generalization failure. The DIODE AbsRel reflects metric scale miscalibration; the DA-2K accuracy reflects the preservation of geometric depth structure.

Three factors contribute to the observed metric gap, each traceable to a structural difference between DIODE and the training curriculum. The first and most significant factor is sensor modality mismatch. The training curriculum uses structured-light and active stereo sensors (Kinect v1, Kinect v2, Asus Xtion, Intel RealSense), all of which produce depth maps with comparable noise profiles and spatial coverage. DIODE is collected using a FARO Focus S350 survey-grade laser scanner [40], a high-precision terrestrial scanning instrument that produces depth maps of substantially higher density and dynamic range than the structured-light sensors used during curriculum training. The measurement principle, noise characteristics, spatial coverage patterns, and valid-depth distributions of a survey-grade laser scanner differ fundamentally from those of the Kinect and RealSense sensors that the metric head was calibrated on during Stages 3 and 4 of the curriculum. This introduces a systematic scale offset that is not corrected without sensor-specific adaptation. Notably, because DIODE covers both indoor and outdoor scenes within the same sensing setup, its indoor split also contains scene diversity and depth range characteristics that extend well beyond the furniture-centric, controlled indoor environments of the training data, compounding the sensor-level mismatch with a scene-level one. The second factor is depth range distribution shift. The curriculum operates within [0.1,10.0] m on NYU Depth V2 and [0.1,8.0] m on SUN RGB-D, both of which capture furniture-centric indoor scenes concentrated in the near-field depth range. DIODE captures more spatially open environments, including corridors, open-plan offices, and rooms with higher ceilings, where valid depth values are distributed across a wider range and the depth histogram is shifted relative to the training distribution. Even with GT clipped to [0.1,10.0] m for evaluation, the within-range distribution differs sufficiently from the training data to degrade metric head calibration. The third factor is scene content and illumination diversity. NYU Depth V2 and SUN RGB-D are collected in residential and academic environments with predominantly artificial and mixed lighting. DIODE’s indoor split encompasses a broader range of room types and illumination conditions. While the ViT-S encoder’s DINOv2-pretrained features are substantially robust to illumination variation, the metric head is a shallow adaptation layer calibrated on the narrower training distribution and is more sensitive to out-of-distribution scene statistics.

The current curriculum was designed for the specific target domain of SUN RGB-D, and its zero-shot behavior on DIODE reflects that design choice transparently. The results confirm that the curriculum preserves strong geometric priors, as evidenced by the DA-2K pairwise accuracy and the $\delta_{2}$/$\delta_{3}$ recovery on DIODE, while metric scale calibration remains sensor-specific. Addressing the sensor modality gap is the highest priority direction for extending the approach to broader deployment scenarios.

## Appendix H. Surface-to-Centroid Gap: Supplementary Analyses

Supplementary per-class, distributional, and reference-definition analyses supporting Table 17 in Section 5.3. Across the SUN RGB-D dataset, the median offset between the two definitions is 0.414 m, and in 85% of instances the centroid lies behind the visible surface. The magnitude of this offset exceeds the MAE of several evaluated models, so evaluation outcomes can vary substantially depending on the reference definition chosen. Per-class medians range from 0.149 m (toilet, a low-depth object mounted close to a wall) to 0.695 m (bed, a volumetric object with large depth extent when viewed from the side). The offset also scales with absolute depth, rising from 0.26 m for objects at 0–2 m to 0.85 m for objects beyond 4 m, reflecting geometric foreshortening at greater range. A per-sensor breakdown across the four SUN RGB-D sensors, reported in Table A3, shows median offsets clustered within a 0.096 m band, confirming that the gap reflects the annotation convention rather than a sensor-specific measurement artifact. Figure A7 visualizes the full instance-level distribution of ΔZ. Per-sensor surface-to-centroid offsets appear in Appendix Table A3.

The choice between surface and centroid definitions changes the reference value itself. Across the 18,352 canonical-class test instances in Table 17, the median offset between surface distance (median depth over 2D box) and centroid distance (3D box center) is 0.414 m. The final system achieves MAE = 0.1286 m under surface evaluation (Depth Anything V2 ViT-S, hole-filled GT depth, mean aggregation) and 0.322 m under centroid evaluation, demonstrating that the choice of reference definition substantially inflates the reported error independently of model quality. Thus, cross-definition comparisons conflate annotation geometry with model quality.

We adopt a surface-based distance definition (mean depth over the predicted 2D bounding box). This aligns with our depth estimator’s training objective and allows evaluation using only registered depth maps, without 3D bounding box annotations. Table 17 reports the offset to centroid-based definitions for cross-comparison. The same-box protocol is therefore defined for surface-based distances derived from dense depth maps. Extension to centroid-based distances from amodal 3D bounding boxes would require additional geometric correction to account for the surface-to-centroid offset, which has a dataset-level median of 0.414 m across the ten canonical SUN RGB-D classes as quantified in Table 17.

## Appendix I. Per-Sensor Surface-to-Centroid Offset

Per-sensor breakdown of the surface-to-centroid offset ΔZ on SUN RGB-D (Table A3).

**Table A3:** Per-sensor breakdown of the surface-to-centroid offset ΔZ on SUN RGB-D. The narrow range of medians across four heterogeneous depth sensors confirms that the discrepancy is definitional rather than sensor-specific.

Sensor	N (inst.)	$\Delta Z_{\mathrm{med}}$ (m)	$\Delta Z_{\mathrm{mean}}$ (m)	$\Delta Z_{p95}$ (m)
Asus Xtion	4364	0.370	0.442	1.07
Kinect v1	2326	0.454	0.529	1.25
Kinect v2	9634	0.438	0.528	1.31
RealSense	2028	0.358	0.457	1.20
